# CilioGenics: an integrated method and database for predicting novel ciliary genes

**DOI:** 10.1093/nar/gkae554

**Published:** 2024-07-11

**Authors:** Mustafa S Pir, Efe Begar, Ferhan Yenisert, Hasan C Demirci, Mustafa E Korkmaz, Asli Karaman, Sofia Tsiropoulou, Elif Nur Firat-Karalar, Oliver E Blacque, Sukru S Oner, Osman Doluca, Sebiha Cevik, Oktay I Kaplan

**Affiliations:** Rare Disease Laboratory, School of Life and Natural Sciences, Abdullah Gul University, Kayseri, Turkiye; Department of Molecular Biology and Genetics, Koc University, Istanbul 34450, Turkiye; Rare Disease Laboratory, School of Life and Natural Sciences, Abdullah Gul University, Kayseri, Turkiye; Rare Disease Laboratory, School of Life and Natural Sciences, Abdullah Gul University, Kayseri, Turkiye; Rare Disease Laboratory, School of Life and Natural Sciences, Abdullah Gul University, Kayseri, Turkiye; Istanbul Medeniyet University, Science and Advanced Technologies Research Center (BILTAM), 34700 Istanbul, Turkiye; School of Biomolecular and Biomedical Science, Conway Institute, University College Dublin, Belfield, Dublin 4, Ireland; Department of Molecular Biology and Genetics, Koc University, Istanbul 34450, Turkiye; School of Medicine, Koç University, Istanbul 34450, Turkiye; School of Biomolecular and Biomedical Science, Conway Institute, University College Dublin, Belfield, Dublin 4, Ireland; Istanbul Medeniyet University, Science and Advanced Technologies Research Center (BILTAM), 34700 Istanbul, Turkiye; Goztepe Prof. Dr. Suleyman Yalcin City Hospital, Istanbul, Turkiye; Izmir University of Economics, Faculty of Engineering, Department of Biomedical Engineering, Izmir, Turkiye; Rare Disease Laboratory, School of Life and Natural Sciences, Abdullah Gul University, Kayseri, Turkiye; Rare Disease Laboratory, School of Life and Natural Sciences, Abdullah Gul University, Kayseri, Turkiye

## Abstract

Uncovering the full list of human ciliary genes holds enormous promise for the diagnosis of cilia-related human diseases, collectively known as ciliopathies. Currently, genetic diagnoses of many ciliopathies remain incomplete (1–3). While various independent approaches theoretically have the potential to reveal the entire list of ciliary genes, approximately 30% of the genes on the ciliary gene list still stand as ciliary candidates (4,5). These methods, however, have mainly relied on a single strategy to uncover ciliary candidate genes, making the categorization challenging due to variations in quality and distinct capabilities demonstrated by different methodologies. Here, we develop a method called CilioGenics that combines several methodologies (single-cell RNA sequencing, protein-protein interactions (PPIs), comparative genomics, transcription factor (TF) network analysis, and text mining) to predict the ciliary capacity of each human gene. Our combined approach provides a CilioGenics score for every human gene that represents the probability that it will become a ciliary gene. Compared to methods that rely on a single method, CilioGenics performs better in its capacity to predict ciliary genes. Our top 500 gene list includes 258 new ciliary candidates, with 31 validated experimentally by us and others. Users may explore the whole list of human genes and CilioGenics scores on the CilioGenics database (https://ciliogenics.com/).

## Graphical Abstract

**Graphical Abstract:**
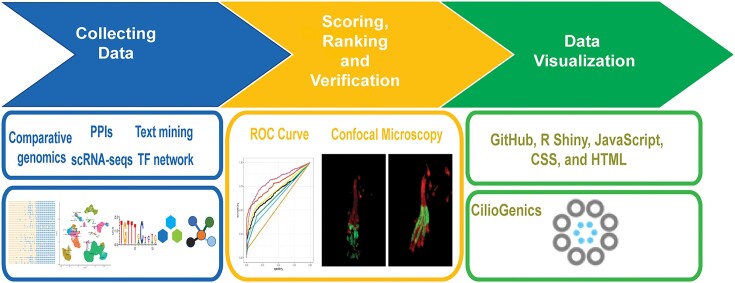

## Introduction

Cilia are cellular organelles that protrude from the surfaces of most cells and play critical roles in cellular motility, sensation, and embryonic development (1). Based on the organization of their microtubule-based axonemal core (presence or absence of a central pair of microtubules) and their motility behavior, cilia are subdivided into two types: motile and non-motile (primary cilium). Different types of cilia perform distinct functions. Mucus clearance, for example, requires the coordinated beating of multiple motile cilia on epithelial cells in respiratory organs, whereas the non-motile cilium is needed for chemical, thermal, and mechanical sensations (2). Functional and structural abnormalities of cilia lead to diseases such as Bardet-Biedl Syndrome (BBS), Joubert syndrome (JS) and Nephronophthisis (NPHP), collectively known as ciliopathies. These conditions manifest with symptoms such as cystic kidneys, retinal degeneration, retinitis pigmentosa, obesity and intellectual disability, resulting from defects in multiple organs and tissues (3–6). Many signaling pathways, including Hedgehog, Wnt, Notch, Hippo and PDGF, essential for cell/tissue formation and homeostasis, have been linked to primary cilia, and some of their components are enriched in primary cilia (7).

Because of the significance of cilia for human health, many researchers have been trying to identify the parts of this tiny and complex organelle. A variety of approaches have been used to determine the protein composition of cilia, leading to the identification of over 600 ciliary genes and many candidate ciliary genes. These approaches include genomic comparisons of ciliary and non-ciliary organisms, the presence or absence of ciliary gene-specific transcription factor (TF) binding sites such as the X-box motif, an increase in gene expression during cilia assembly, functional genomics (e.g. RNAi and CRISPR screening), clinical studies, co-expression of ciliary genes within specific tissues, proximity labeling, and proteomics (8–56). The constant discovery of new ciliary genes implies that these independent methods are inadequate to uncover all ciliary genes, even though they have assisted in the discovery of many ciliary genes. This is especially true for comparative genomics, which reveals new ciliary genes based on being present in organisms with cilia but not in organisms that do not have cilia.

Because cilia are highly specialized organelles with specific roles that are required in some cell types but not all in humans or other species, cilia-enriched genes are more likely to be expressed in ciliated cells than in nonciliated cells. A single-cell RNA sequencing (scRNA-seq)-based method, which would reveal gene expression differences among cell types, would overlook genes expressed in all cell types but required for cilia assembly and/or function. The main problem is that all of these independent approaches have produced far too many ciliary candidate genes, and the real question is whether there is a better method for discovering new ciliary genes that outperform others.

Here, we develop an integrated method called CilioGenics, which integrates several independent methods, including comparative genomics, scRNA-seq analysis, gene regulatory networks, protein–protein interactions (PPI) and text mining. Our unbiased methods involve interrogating the entire human genome and assigning a score to each gene for its potential to be a ciliary gene, thus aiming to address the ongoing challenge of identifying a comprehensive list of ciliary genes in humans. The anticipated improvement with CilioGenics contributes to the efficiency of ciliary gene identification, yielding a more detailed and comprehensive compilation of ciliary genes. Importantly, the integrated CilioGenics method is superior to any single method or CiliaCarta, allowing us to confidently identify novel ciliary genes. We have successfully validated the ciliary localization of numerous newly identified genes, such as *ZC2HC1A, ZNF474, WDR54*, *TMEM145* and *TTC39C*. To facilitate further exploration, all data is accessible on the CilioGenics website (https://ciliogenics.com/).

## Materials and methods

### scRNA-seq analysis of human tissues and single-cell RNA sequence analysis of *C. elegans*

The human lung scRNA-seq raw data were retrieved from the Gene Expression Omnibus (www.ncbi.nlm.nih.gov/geo). The following accession number was used to download the raw data: (GSE122960) (48), followed by analysis with the Seurat R package (86). Default settings were used, and codes for analysis can be found at https://github.com/thekaplanlab/CilioGenics_Analysis. The pre-analyzed data for the human lung, pancreas, trachea, brain, and retina scRNA-seqs were obtained as an RDS file (59–64). For *C*. elegans scRNA-seq, the RDS file data was downloaded from https://www.cengen.org/ and NCBI (17,57).

We performed differential expression analysis between ciliated and other cellular clusters using the Seurat R package with default settings (SeuratObject 4.0.1 and Seurat 4.0.1) (86). Codes for analysis can be found at https://github.com/thekaplanlab/CilioGenics_Analysis. In summary, established ciliated cells comprised group 1, while all other cells comprised group 2. For every gene, the difference between the percentage of cells expressing it in group 1 and the percentage in group 2 was calculated and normalized for each sample. Consequently, higher scores were assigned to genes exclusively expressed in ciliated cells. In *C. elegans* data, this difference was generally small, and therefore the distribution of data was too skewed. Therefore, for *C. elegans* data, logs of these scores were taken before normalization. Supplementary Tables S1 and S2 contain ciliary candidate gene names from *C. elegans* scRNA-seq and human scRNA-seq, respectively. Moreover, the versions of all R packages used are outlined in a file named ‘Package Versions.’

### BLAST analysis for 72 organisms and clustering

Phylogenetics-based scoring of all genes was performed using proteomics data available on NCBI. The proteomes of 72 organisms were downloaded. The longest protein transcript from each gene of the human genome was selected, and the proteome of the remaining 71 organisms was the Basic local alignment search tool protein (BLASTp) against the selection of human proteins (87). The list of organisms with download links is provided in Supplementary Table S11 (Supplementary Table S11). The results were sorted by the bit scores, and only the transcripts from each gene with the highest bit scores were kept. Then, the genes with bit scores lower than 50 and *P*-values higher than 0.001 were filtered out. The remaining gene list from each organism was considered to have homologous genes in *H. sapiens*. For clustering, first, a dissimilarity matrix was generated using the ‘daisy’ R function (Cluster package version 2.1.4) with the Gower metric. Then hierarchical clustering was applied to the dissimilarity matrix to generate a tree. To efficiently separate data into clusters, the resulting trees were divided into 40 clusters using the ‘cutree’ function. Every cluster underwent scoring, and only two clusters (Cluster 31 and Cluster 37) were pinpointed as harboring genes with orthologs predominantly found in ciliated organisms. All genes within the two highest-scoring clusters received a score of 1, while all others were assigned a score of 0 (determined through visual inspection). The CilioGenics website provides users with access to the names of genes in each cluster (https://ciliogenics.com/).

### Protein–protein interactions (PPI) analysis

The protein–protein interaction (PPI) data were sourced from three primary databases: IntAct (https://www.ebi.ac.uk/intact/), BioGRID (https://thebiogrid.org/), and HuRI (http://www.interactome-atlas.org/) (77–79). This study specifically includes interaction data involving the organisms, such as *Homo sapiens, Mus musculus, Drosophila melanogaster* and *Caenorhabditis elegans*. In the case of IntAct, interactions lacking a PPI score (MIscore) were excluded. MIscore is a scoring system that is utilized to score the degree of confidence of an interaction (88). From BioGRID, only interaction data about *H. sapiens, M. musculus, D. melanogaster* and *C. elegans* were incorporated. Data exclusively from *H. sapiens* was utilized from HuRI. To enhance consistency, all identifiers, including gene IDs and UniProt IDs, were converted to HGNC gene names (HUGO Gene Nomenclature Committee). Subsequently, gene names originating from non-human organisms were converted to their human homologs using the Alliance Genome (89). In the combination of all three datasets, redundant interactions were eliminated. Interactions were deemed redundant if both interactors and publication identifiers were identical.

To quantify interactions, PPI scores were computed for each gene, employing a modified version of the MIscore. This considered factors in the detection method and interaction type, such as direct interaction, biochemical, protein complementation assay, post-transcriptional interference, genetic interaction, and the physical association, as well as the number of publications. Here are the scores: MI:0013: biophysical: 1, MI:0090: protein complementation assay: 0.66, MI:0254: genetic interference: 0.10, MI:0255: post-transcriptional interference: 0.10, MI:0401: biochemical: 1, MI:0428: imaging technique: 0.33, MI:0208: genetic interaction: 0.1, MI:0403: colocalization: 0.33, MI:0914: association: 0.33, MI:0915: physical association: 0.66, MI:0407: direct interaction: (i) Notably, we omitted the consideration of publications in the scoring process to prevent bias towards well-established ciliary genes. The method score and score type were calculated as per previous descriptions, and an average score was designated as the PPI score. These PPI scores were then utilized to calculate the Ciliary Genes Interaction (*cgi*) score using the following formula, where ***Ln*** represents the total number of interactions of the gene of interest and *Lg* is the geometric average of PPI scores between the gene and known cilia genes.


\begin{eqnarray*}cgi = log\left( {Ln/5 + 1} \right)\;*\;Lg\end{eqnarray*}

### Transcription factor (TF) target interaction prediction

The regulome data were obtained from PANDORA (http://120.79.46.200:81/Pandora/). Specifically, only TF (Transcription Factor)-target regulations from ciliated cells were utilized (70). Mouse gene names were converted to human HGNC names. Furthermore, the predicted FOXJ1 regulatory network gene list was taken from Mukherjee et al. and combined with the other list (90). The regulome data encompassed information from 11 different organisms. Genes regulated by TFs (RFX2, RFX3, MYB, GLIS3, JAZF1, SOX5 and TOX) were assigned scores based on the number of organisms in which the TF binds to that gene (71–76). For FOXJ1, the score was determined by the frequency of this interaction in the data. If FOXJ1 could bind to different parts of the same gene, the score was assigned based on the number of binding occurrences. The next step involved taking the logarithm of these scores, adding 1 to each score (log (score + 1)), and then summing the scores for each gene. Finally, the logarithm of the resulting sum was taken (log (sumOfScores + 1)). This final score represents the motif data. The gene names coming from TF analysis are listed in Supplementary Table S6.

### Text mining (protein atlas)

The protein atlas website was scanned, and the gene entries having at least one of the ‘cilia, cilium, flagella, flagellum, and/or centrosome’ words were filtered (84). Any entry containing the ‘centrosome’ word is given 0.25, while the ‘flagellum’ word awards the gene a score of 1. If ‘cilia’ and ‘cilium’ words were accompanied by ‘positivity’ or ‘stain’, then it is assumed that they show ciliary localization rather than expression, thus the gene was awarded by 1, otherwise 0.5. The total scores for each gene were calculated and normalized to yield a score between 1 and 0 for each gene. Supplementary Table S8 contains a list of all gene names discovered using text mining.

### Ciliary gold standard genes (GSCGs) and negative ciliary genes (NCGs)

Nevers and colleagues presented the negative ciliary genes, whereas the updated ciliary gold standard ciliary genes were used (5,65). Gold standard ciliary genes (GSCGs) are listed in Supplementary Table S3, whereas negative ciliary genes (NCGs) are listed in Supplementary Table S4.

### Collection of publications

The names, types of organisms, web links and other information about the papers and tables that contained the list of ciliary candidate genes were gathered from all cilia-related publications and reported in Supplemental Table S10.

### Creation of the CilioGenics database

For the comprehensive cilia database, Shiny (an R package, version 1.6.0) was used to develop a database called CilioGenics. To search for a gene in the CilioGenics database, users have a variety of options, including human gene names, synonyms, and gene names and gene IDs from various model organisms, including *Mus musculus*, *Rattus norvegicus*, *Danio rerio*, *C. elegans, Drosophila melanogaster, Saccharomyces cerevisiae, Chlamydomonas reinhardtii* and *Tetrahymena thermophila*. We retrieved orthology gene lists between human and *C. reinhardtii*, as well as human and *T. thermophila* from https://omabrowser.org/ (91), and between human and the remaining species from Alliance of Genome Resources (89). As a part of our previous work, we generated the *C. elegans* orthologs of human genes (89,92).

### Strains maintenance

The *C. elegans* wild-type isolate strain (N2) were raised on the previously described Nematode Growth Medium (NGM) (93).

### Generation of translational and transcriptional transgenic strains

The pPD95.67 plasmid, a modified *C. elegans* expression vector, was used to clone the 500 base pairs (bp) of the gene promoter, followed by 1542 bp of the cDNA for C15A7.2 (*tmem-145*) directly in front of the GFP. The length of a gene's promoter was limited to 500 base pairs; however, the length of each cDNA was as follows: 396 bp for cDNA of M153.4 (znf-474), 1044 bp for cDNA of F39H12.2 (*wdr-54*), and 969 bp for cDNA of T03G11.3 (*zchc-1a*). SphI and AgeI restriction enzymes were used in the cloning. The resulting plasmids were as follows: *wdr-54promoter::WDR-54::GFP (500 bp promoters)*; *tmem-145promoter::TMEM-145::GFP (500 bp promoters)*; *znf-474promoter::ZNF-474::GFP (500 bp promoters)*; and *zchc-1a::ZCHCC-1A::GFP (500 bp promoters)*. Except for *tmem-145*, all of the other genes and 1380 bp of cDNA of K10G6.4 (*cank-26*) were cloned between 500 base pairs of the long *arl-13* promoter (a cilia-specific promoter) and GFP. The following plasmids were produced: *arl-13promoter::WDR-54::GFP (500 bp promoters); arl-13promoter::ZNF-474::GFP (500 bp promoters); arl-13::CANK-26::GFP (500 bp promoters)*; and *arl-13a::ZCHC-1A::GFP (500 bp promoters)*. The *TTC-39c::GFP* (C32D5.6) Fosmid was purchased from Source BioScience. For transcriptional strains, the 1000 bp promoter regions of *zchc-1a*, *tmem-145*, and *wdr-54* were cloned by SunyBiotech, China and inserted into the ppd49.83-NLS-GFP vector. Unless stated, a microinjection of 50 ng/μl RF4 (Roller selection marker) at a dosage of 5–25 ng/μl was performed for each construct. The resulting transgenic strains were crossed into Intraflagellar Transport 140 (IFT-140):mCherry (known as CHE-11), an IFT-A component used as a ciliary marker.

#### Genetic crossing

Standard genetic crossing techniques were used to generate mutants: transgenes lines. PCR using primers that flank deletions were used to follow the *zchc-1a(syb849), mem-145(tur009), wdr-54(syb1005), znf-474(tur006), ttc-39c(syb771), cank-26(gk567)* deletion mutations (Supplementary Table S12).

#### Dye-filling assay

In the first step, *C. elegans* worms were incubated in a solution of red fluorescent lipophilic dye DiI solution (Invitrogen) diluted 1:200 with M9 buffer (200 μl) for 45 min. The lipophilic and red fluorescent DiI specifically labels the ciliated sensory neurons (Amphid/Phasmid cells). Following incubation, worms were transferred to seeded nematode growth medium (NGM) plates for recovery for one hour. Finally, worms were mounted on slides for visualization using a fluorescence compound microscope with a 20x objective. The acquired images (shown in the accompanying figure) reveal the uptake of DiI by the ciliated Amphid/Phasmid cells.

### Behaviour assays

*Chemosensory assays:* A chemotaxis assay tested *C. elegans* attraction to isoamyl alcohol (1:100 dilution in 95% ethanol). 60mm plates were divided into attractant (alcohol + levamisole), control (ethanol + levamisole), and worm placement region. Young adult worms were rinsed from non-contaminated seeded plates three times using M9 buffer and once with deionized water. Washed young adults (∼100) were added and allowed to disperse (30 min) once excess liquid was removed by Kim wipe. Chemotaxis index ((# at attractant – # at control)/total worms) quantified the response. The chemotaxis index was calculated at the 30-min mark.

*Osmotic avoidance behavior assay:* Osmotic avoidance was performed on 60 mm unseeded plates. 1 day synchronized adult worms were left on unseeded plates for an hour. Then 5 worms were placed in the center of an 8 M glycerol ring. Then the number of worms inside the ring were counted after 5 min. *osm-3(p802)* worms were used as a negative control as they are deficient in osmotic avoidance behavior, N2 worms were used as a positive control.

### Confocal microscopy

The Z-stack images, (a Plan ApoChromat 63×/1.40 NA lens and 0.14 μm intervals between images), were acquired through the Zeiss LSM900 confocal microscope with Airyscan 2. The Zeiss microscopy was managed by the ZEN Blue Edition software. Briefly, microscope slides were prepared with 2–3% agarose, and worms were placed onto the agarose pad. To immobilize the worms, 10 mM levamisole was applied. Subsequent image analysis was performed using ImageJ (NIH) software (94).

### Generation of mutant strains with CRISPR/Cas9

After designing sgRNAs for *tmem-145* and *znf-474* using ChopChop, sgRNAs were cloned into the empty sgRNA vector (pRB1017, Addgene: #59936) (95,96). Each gene had three independent sgRNAs designed. Subsequently, these sgRNAs were microinjected into the wild-type (N2) utilizing a Zeiss Axio Vert.A1 inverted microscope equipped with DIC optics and a Narishige Micromanipulator MMO-4. P0s were subjected to F1 selection and PCR genotyping. Upon the finalization of the F2 mutants, they were sent for Sanger sequencing. Two mutants, namely *tmem-145(tur009)* and *znf-474(tur006)*, were successfully generated. The *znf-474(tur006)* mutant contains a 678-base pair deletion, eliminating 310 bp from the majority of exon I and the entire exon II, along with a 54 bp segment of exon III, resulting in the generation of a stop codon. On the other hand, *tmem-145(tur009)* showed two deletions in the gene structure: a 1662-base pair deletion, removing a portion of exon I up to exon VIII, and a 449-base pair deletion, removing exon X and a portion of exon XI. The mutant for *cank-26*(gk567) was obtained from CGC, and three mutants [wdr-54*(syb1005), zchc-1a(syb849)* and ttc-39c*(syb771)*] were generated by SunyBiotech, China. These strains were crossed into GFP-tagged ciliary strains, including *ift-74(cas499[ift-74::gfp])*,*II., osm-6::gfp, and che-3(cas443 [gfp::che-3]). I*. SgRNA sequences, primers and strains can be found in Supplementary Table S12 (Supplementary Table S12).

### ROC curve (receiver operating characteristic curve)

The accuracy of each method was determined using a randomly generated test and trained gene lists, leading to the generation of a confusion matrix. The confusion matrix (cilia vs non-cilia) was used to calculate F1 scores for each approach.


\begin{eqnarray*}{\mathrm{Precision\;}} = \;TP\;/\;\left( {TP + FP} \right)\end{eqnarray*}

\begin{eqnarray*}{\mathrm{Recall}}:{\mathrm{\;}} = \;TP\;/\;\left( {TP + FN} \right)\end{eqnarray*}

F1 Score = 2

F1 Score = 2× Precision x Recall/Precision + Recall = 2TP/2 TP + FP + FN

Number of true positives (TP)

Number of false positives (FP)

Number of false negatives (FN)

F1 scores were determined for each method. With the help of F1 scores, the CilioGenics scores (sum scores) for each gene were calculated. The precision of the computed scores was assessed by generating ROC–AUC curves for each method, including the combined approach CilioGenics and CiliaCarta. ROC curves were constructed by plotting the true positive rate against the false positive rate at varying thresholds.

### Cell culture

hTERT RPE-1 (ATCC-CRL-4000) cells were maintained in DMEM: F-12 media (Gibco, 11320033) supplemented with 10% FBS (Gibco, 26140079) and 1% Pen-Strep (Gibco, 15070063) at 37°C in 5% CO_2_ (97). Human telomerase immortalized retinal pigment epithelium cells (hTERT-RPE, ATCC and CRL-4000) are used for shRNA. Dulbecco's modified Eagle's medium DMEM/F12 50/50 medium (Pan Biotech, Vienna, AUT), 10% FBS (Life Technologies, Carlsbad, CA, USA), 1% penicillin–streptomycin (Gibco, Thermo Fisher Scientific) have been used for cell culture medium.

### Immunostaining

Cell Imaging—hTERT RPE-1 cells were seeded onto sterile 24-mm polylysine-d-coated coverslips in a 6-well plate, maintained in 10% FBS for 24 h, and later switched to serum-free medium for 48 hours to induce ciliogenesis.

Serum-starved hTERT RPE-1 cell on poly-d-lysine-coated glass coverslips were washed twice with cell-washing solution (CWS; 2.6 mM KCl, 137 mM NaCl_2_, 10 mM Na2HPO4, 1.8 mM KH_2_PO_4_). Then cells were fixed with 4% paraformaldehyde and 4% sucrose in CWS for 10 min. Each well was washed three times with CWS and permeabilized by a 5-min incubation with 0.2% Triton X-100 in CWS. Each coverslip was blocked for 60 min. of incubation with 10% normal goat serum (Invitrogen, 50197Z) in CWS. Cells were incubated with anti-WDR-54 antibody (1/100, Invitrogen, PA5-62806) and anti-acetylated tubulin (1/150, MilliporeSigma, MABT868) in 1% BSA-containing CWS. Goat anti-rabbit AlexaFluor488 and anti-mouse Alexa-Fluor594 (Invitrogen, A32731, A32742) secondary antibodies were diluted by 1/500 into a 1% BSA-containing cell washing solution. All antibody dilutions were centrifuged at 10 000 × g for 10 min before use. The nucleus was stained with 1 mg/ml DAPI at the last washing step for 5 min. Slides were then mounted with glass coverslips by using an anti-fade reagent (Invitrogen, P36930) (98).

### qRT-PCR

RNA was isolated from RPE1-shControl, RPE1-shWDR54, RPE1-shZC2HC1A cell lines using NucleoSpin RNA kit (Macherey-Nagel). cDNA synthesis was performed using iScript cDNA synthesis kit (Bio-Rad). qRT-PCR was done with GoTaq qPCR Master Mix (Promega), all according to manifacturer's protocols.

### shRNA expressing cell line generation

shRNAs were cloned into PLKO.1 and made by cotransfection of packaging and envelope vectors in HEK293T cells. After 48 h incubation, supernatant was collected and filtered using 0.22 μm filters (Millipore). For transduction, RPE1 cells were plated to 12-well plates and infected with viruses. Two days after transduction, cells were incubated in complete medium containing 10 μg·ml^−1^ puromycin (Invivogen). Resulting pool of cells were used for assays.

Plasmid to generate shRNA lines: pLKO.1 (Invitrogen, Waltham, MA, USA). Oligonucleotide sequences to generate shRNA lines in PLKO.1 backbone:

**For WDR54: 5′ to 3′**

Forward: ccggCCAGATGCCAATCACAGACATggatccATGTCTGTGATTGGCATCTGGtttttg

Reverse: aattcaaaaaCCAGATGCCAATCACAGACATggatccATGTCTGTGATTGGCATCTGG

**For ZC2HC1A: 5′ to 3′**

Forward: ccggCCACCAAAGAAACCATCTAATggatccATTAGATGGTTTCTTTGGTGGtttttg

Reverse: aattcaaaaaCCACCAAAGAAACCATCTAATggatccATTAGATGGTTTCTTTGGTGG

Primer sequences for qRT-PCR:

**For GAPDH: 5′ to 3′**

Forward: GGATTTGGTCGTATTGGG

Reverse: GGAAGATGGTGATGGGATT

**For WDR54: 5′ to 3′**

Forward: ACGAACCAGGAGTCAGGATGT

Reverse: GCCAAAATACGTGAGGTTGCG

**For ZC2HC1A: 5′ to 3′**

Forward: GGCAAAACTGTTGTAGGTGTTCC

Reverse: TGGAGGGCCTAACTTGTCCA

**Primary Antibodies used:**

1:2000, mouse IgG1 anti polyglutamylated-tubulin (GT335, AG-20B-6020, Adipogen, San Diego, CA, USA).

1:2000, mouse IgG2b anti acetylated tubulin (clone 6-11B, sc-23950, Santa Cruz Biotechnology).

1:2000, rabbit anti Arl13b (17711-1-AP, ProteinTech).

**Secondary antibodies used:**

1:2000 Invitrogen Goat anti rabbit IgG 488 Alexa Fluor 1971418.

1:2000 Invitrogen Goat anti mouse IgG1 Alexa Fluor 568 A21124.

1:2000 Invitrogen Goat anti-mouse IgG2b Alexa Fluor 633 A21146.

**Microscope used:**

### HCPL APO CS2 63 × 1.4 NA oil objective, Leica SP8 confocal microscope

Quantifications of data are done by using Imagej (NIH, Bethesda, MD, USA), Microsoft Excel, prism 6 (Graphpad, San Diego, CA, USA).

### Statistical analysis:

Two-sided Student's t-test were applied. The graphs with error bars indicate standard deviation (SD). Significance states *P < 0.05, **P < 0.01, ***P < 0.001 and **** *P* < 0.0001.

### Coding and files

The codes used for data analysis and constructing the CilioGenics website may be accessed at the respective GitHub: https://github.com/thekaplanlab/CilioGenics_Analysis.

## Results

### Single-cell RNA sequencing analysis of *C. elegans* reveals novel ciliary candidate genes

**Figure 1.**
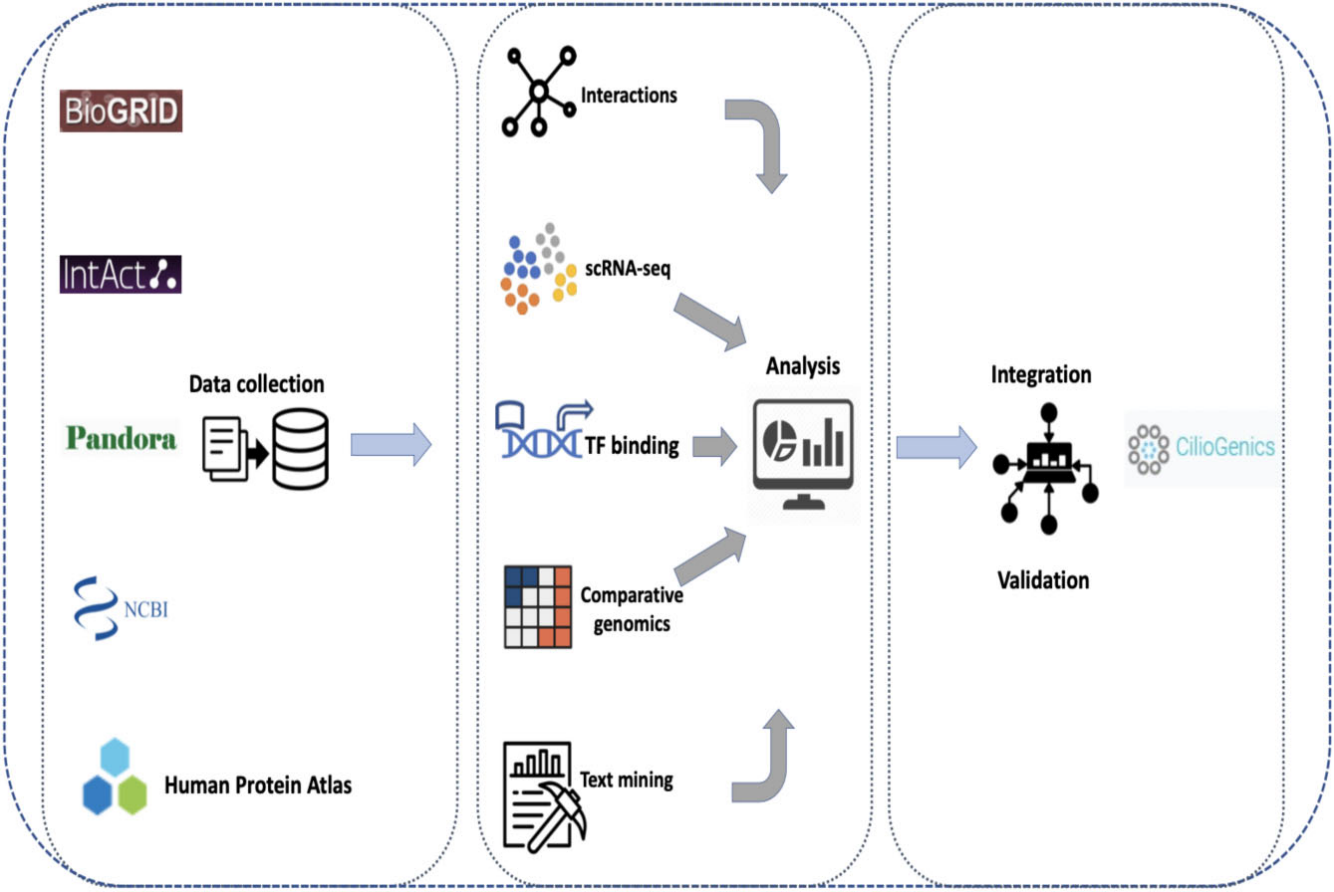
Workflow of CilioGenics. Data from IntAct, BioGRID, HuRI, Pandora and NCBI is gathered, assessed using R scripts, and then integrated with data from other sources.

We integrated multiple datasets to create a novel method for predicting ciliary genes (Figure 1 and Supplementary Figure S1). *C. elegans* has a variety of cell types, including the intestine, muscle, germ cells and neurons (cholinergic neurons and ciliated sensory neurons). Ciliary genes are exclusively expressed in ciliated sensory neurons but not in non-ciliary cells such as the intestine, muscle, germ cells, and non-ciliated neurons (17). Consequently, genes expressed in ciliated sensory neurons but not in other tissues should be identified as ciliary genes. To uncover new ciliary genes, we applied analysis of the single-cell RNA sequencing (scRNA-seq) data from *C. elegans* (17).

**Figure 2.**
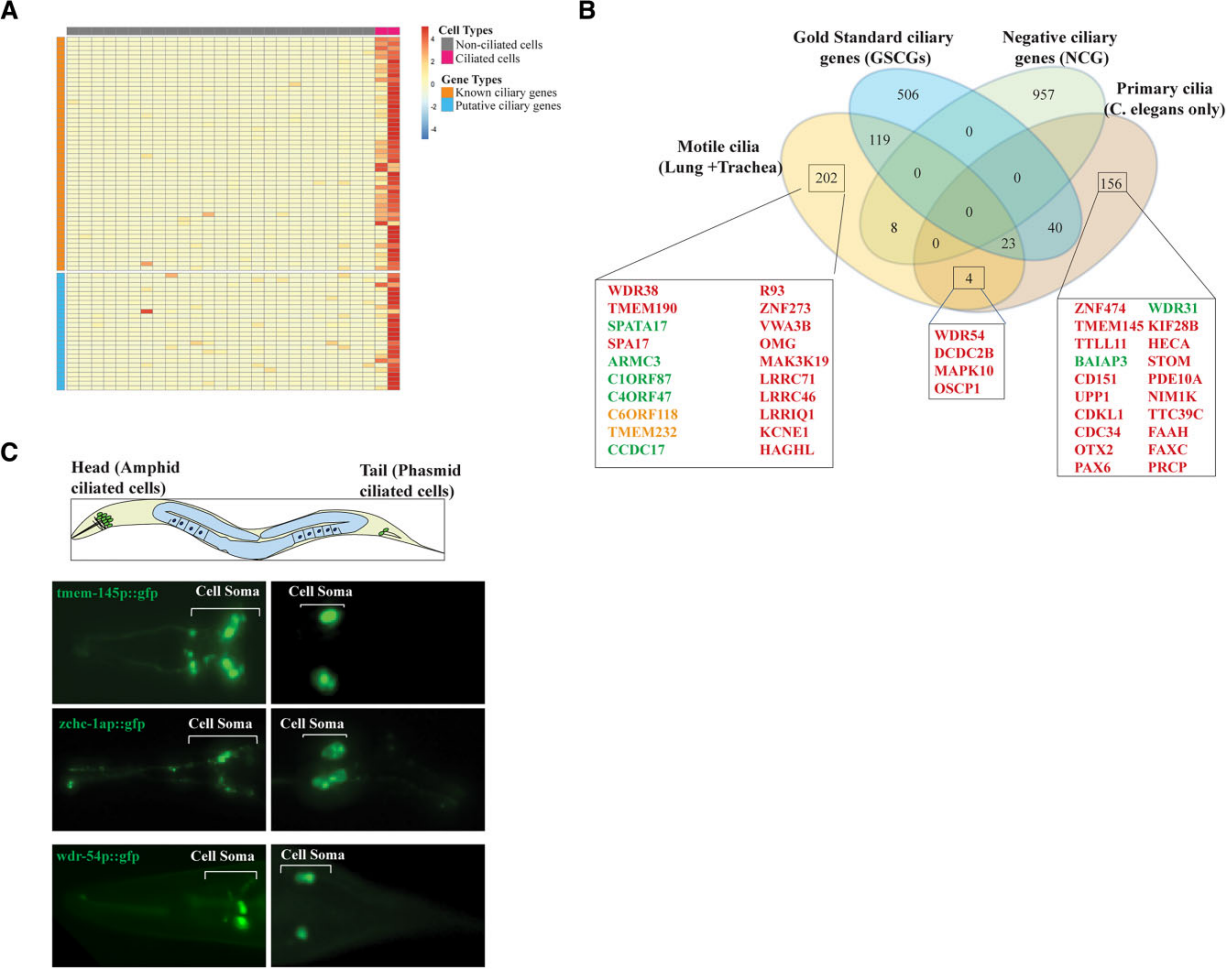
single-cell (sc) RNA-seq of *C. elegans* reveals many ciliary candidate genes. (**A**) The differentially expressed genes in the *C. elegans* scRNA-seq dataset are shown in a representative heatmap. Cells with cilia are denoted by the color red, whereas cells without cilia are denoted by the color gray. (**B**) The gene lists from motile cilia (Lung + Trachea), Gold Standard Ciliary Genes (GSCGs), Negative Ciliary Genes (NCG), and Primary Cilia (*C. elegans* only) were compared and are visualized in the Venn diagram. Ciliary candidate genes are represented in red, while genes encoding proteins localized to cilia and those implicated in cilia biogenesis are depicted in green and orange, respectively (26,39). Please see Figure 8B for WDR54 and ZC2HCA1 localization. (**C**) Shown are drawings of *C. elegans*. The ciliated sensory neurons (green) in the head and tail are depicted. Head and tail fluorescence images show the expression of *wdr-54promoter::gfp, tmem-145promoter::gfp* and *zchc-1apromoter::gfp*. Cell somas (body) of sensory neurons are depicted in white brackets.

Analysis reveals that 1989 *C. elegans* genes appeared to be differentially expressed and specifically enriched in ciliated sensory neurons, including amphid, phasmid, and oxygen sensory neurons, and may be good ciliary candidate genes; however, only 685 have human counterparts, and those without are ignored. We discovered that 379 of the 685 genes are already known ciliary genes, while the rest are classified as putative candidate ciliary genes that we are further investigating (the Cao scRNA-seq list: Supplementary Table S1A). Expectedly, many core ciliary genes, including intraflagellar transport (IFT) components, transition zone (TZ) and BBSome components, exhibit exclusive ciliary cell-specific expressions (Figure 2A), and many previously unknown ciliary genes recapitulate the cilia-specific expression patterns of known ciliary genes. Subsequently, we analyzed the scRNA-seq dataset from the *C. elegans* Neuronal Gene Expression Map & Network (CeNGEN) (57) and then compared the ciliary candidate gene list from the cilia-specific cluster of CeNGEN with the Cao scRNA-seq list. We compiled a ciliary candidate gene list, giving special consideration to genes present in both, as they are more likely to be putative ciliary genes. The combined list has 232 genes, known ciliary genes, and novel ciliary gene candidates, including *TMEM145, WDR31, WDR54* and ZNF474 (Figure 2B and Supplementary Table S1B). Our previous work already confirmed *WDR31* (WD Repeat Domain 31) (*C. elegans T05A8.5*) as a new ciliary gene, and we validated the exclusive expression of *wdr-31* in the ciliated sensory neurons in *C. elegans*, as well as its localization to the cilia and basal body in human cell lines and *C. elegans* (58). Next, we proceeded to confirm the ciliated cell-specific expressions of several putative ciliary candidate genes from our list (Supplementary Table S1A and B). Specifically, we examined the expression patterns of *tmem-145* (corresponding to human *TMEM145* and C. elegans *C15A7.2*), *wdr-54* (human *WDR54* and *C. elegans F39H12.2*), and *zchc-1a* (human *ZC2HC1A* and C. elegans *T03G11.3*) in *C. elegans*. Our findings revealed that these genes exhibit exclusive expression within the ciliated sensory neurons of *C. elegans*, further indicating their potential as ciliary candidate genes (Figure 2C and Supplementary Figure S10).

### Analysis of human lung and trachea scRNA-seq datasets uncovers new ciliary candidate genes

**Figure 3.**
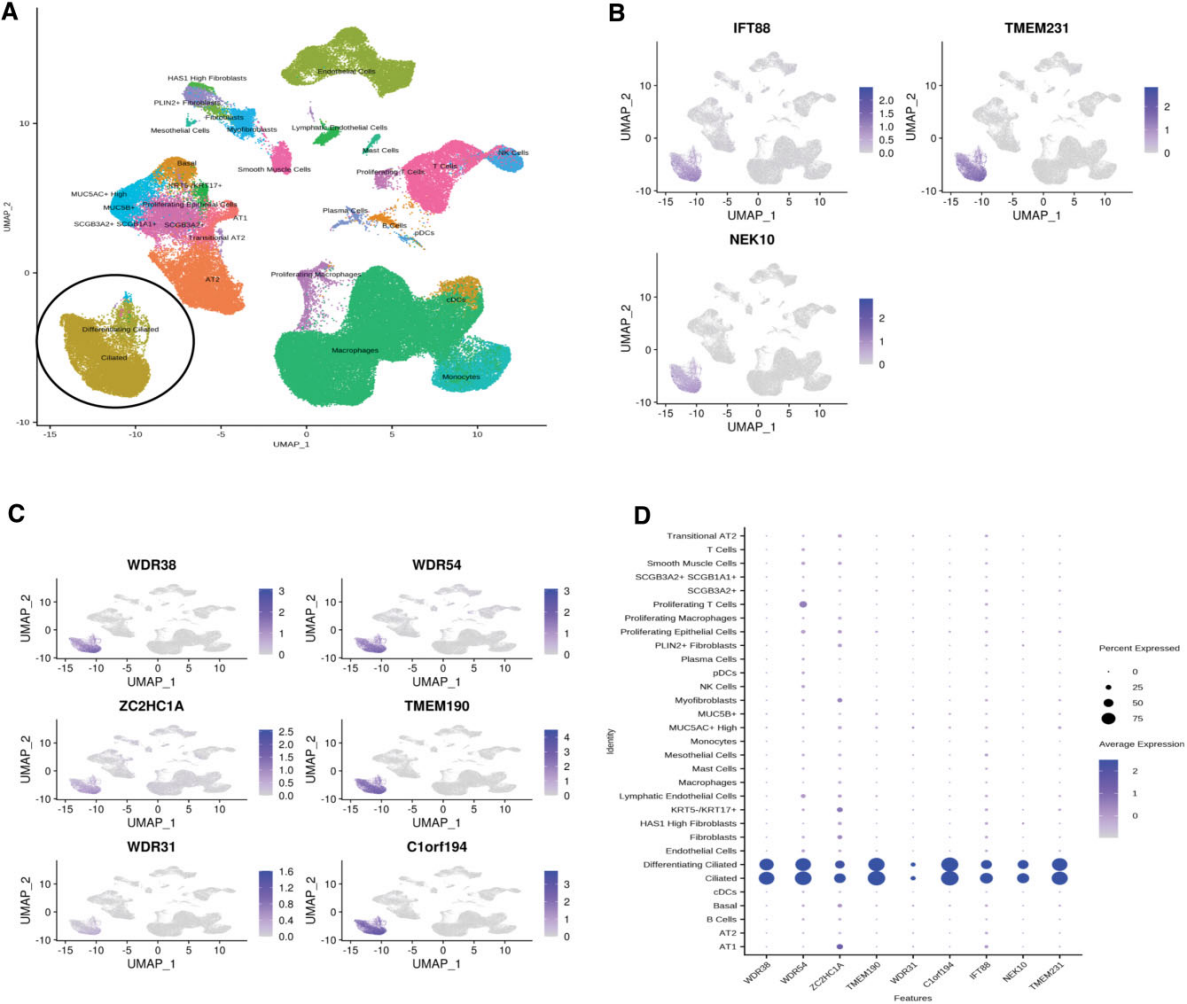
Human lung scRNA-seq reveals many ciliary candidate genes. (**A**) The UMAPs from human lung scRNA-Seq are shown (Habermann study). (**B**) UMAPs of IFT88, TMEM231, and NEK10 from scRNA-seq (Habermann study) are presented. (**C**, **D**) UMAPs and Dotplots of the indicated ciliary candidate genes for scRNA-seq (Habermann study) are displayed.

Subsequently, we turned our attention to the analysis of scRNA-seq data derived from human lung tissue. Given the diverse array of cell types present, including multiciliated epithelial cells (motile cilia), club cells, as well as alveolar type I (AT1) and type II (AT2) cells, the lung serves as an ideal model system for comparing the expression profiles of ciliated and non-ciliated cells. We chose four distinct lung scRNA-seq datasets, analyzed them separately, and compared the ciliary candidate gene list with one another. We identified a cumulative total of 1802 (Reyfman), 1157 (Carraro), 2165 (Habermann), and 966 (Murthy) genes that exhibit exclusive expression within ciliated cells. Among these genes, 1082 (Reyfman), 719 (Carraro), 1017 (Habermann) and 406 (Murthy) emerged as potential candidate ciliary genes (48,59–61). The Reyfman dataset contained 240 known ciliary genes, the Carraro dataset comprised 231 known ciliary genes, the Habermann dataset included 287 known ciliary genes, and the Murthy dataset had 190 known ciliary genes (Supplementary Table S2). We then analyzed the scRNA-seq dataset from the trachea (motile cilia) (62) and identified 518 genes that are potential ciliary candidate genes. Importantly, similar to known ciliary genes, including IFT88 (an IFT gene required for cilia formation), TMEM231 (transition zone gene essential for the ciliary gate controlling), and NEK10 (NIMA Related Kinase 10) (Figure 3A and B), genes previously unknown as ciliary candidate genes, including *WDR38, TMEM190*, *ZC2HC1A* and *C1orf194*, are expressed in ciliated cells, as depicted in dot plots from human single-cell RNA sequencing (Figure 3C and D, Supplementary Figures S2–S4, please visit https://ciliogenics.com/ for other scRNA-seq data). Users may visualize the expression patterns of each gene for *C. elegans* and human scRNA-seq data on the CilioGenics website (https://ciliogenics.com/)

Subsequently, we conducted a comparative analysis of the ciliary candidate genes derived from these five distinct scRNA-seq studies, revealing a set of 356 genes that are shared by all five datasets. This gene list likely contains genes that are specific to motile cilia since the lung and trachea possess motile cilia. Subsequently, we extended our analysis to include scRNA-seq data from tissues with primary cilia (non-motile), including the liver, pancreas, retina, and hypothalamus brain, in an attempt to identify genes unique to primary cilia (Supplementary Figure S5, Supplementary Figure S6A–D); please visit https://ciliogenics.com/).

However, our analysis did not identify a cilia-specific cluster for the tissues analyzed due to the ubiquitous presence of cilia in most cells of these organs (62–64). For instance, the retina has various cell types, such as photoreceptor cells (rods and cones), bipolar cells, ganglion cells, horizontal cells, amacrine cells, and Müller glial cells, all of which possess cilia (65–67). However, we need to note here, in brain scRNA-seq, numerous ciliary genes, such as IFT (intraflagellar transport required for cilia formation) and BBS (Bardet Biedl Syndrome), exhibit exclusive expressions in neurons, but the large number of genes (8497 in total) expressed in neurons resulted in insufficiently specific results to warrant further analysis (Supplementary Figure S6; visit https://ciliogenics.com/).

Given that *C. elegans* possesses non-motile sensory cilia, we compared the ciliary candidate genes (356 genes) identified through four distinct human lung single-cell RNA sequencing (scRNA-seq) and trachea scRNA-seq analyses with the ciliary candidate gene list from *C. elegans* scRNA-seq (233 genes, Supplementary Table S1B). This comparison aimed to unveil genes that are specific to motile cilia and primary cilia (Figure 2B and Supplementary Table S2). The comparison included Gold standard ciliary genes (GSCGs) as well as negative ciliary genes (NCGs) (Figure 2B, Supplementary Table S3, and Supplementary Table S4). The sizes of GSCGs and NCGs are 688 and 965, respectively (55,68). It is noteworthy that human genes without counterparts in *C. elegans* will likely be missed in the analysis. The comparison revealed that 202 genes are specific to motile cilia in the lung and trachea, whereas 156 genes are specific to sensory cilia in *C. elegans* (Figure 2B). The primary ciliary gene list has many known ciliary genes, such as *BBS1, BBS2, BBS4, BBS5, BBS7* and *TTC8*, and IFT genes, as well as putative ciliary genes, such as *ZNF474, TMEM145, BAIAP3*, and *WDR31* (Supplementary Table S1). The motile cilia gene list contains many known ciliary genes, such as *TTC21A, IFT122, IFT140, IFT172, IFT22, IFT27, IFT43, IFT57, IFT80, IFT81* and *IFT88*, as well as putative ciliary genes, such as *WDR38, TMEM190*, *SPA17, RIBC2* and *C1orf194* (Supplementary Table S2). We would expect that the expression of motile cilia-specific genes should be absent in tissues with primary cilia. Consistent with this expectation, the expression of *WDR38* and *TMEM190* is absent in the retina (Supplementary Figure S4C). Both motile cilia and primary cilia gene lists have *WDR54* and *OSCP1* (Figure 2B). We already confirmed that *TMEM-145, WDR54* and *ZC2HC1A* are expressed in the ciliated cells of *C. elegans* (Figure 2C). In addition, independent studies revealed that OSCP1, BAIAP3 and SPA17 localize to cilia (26,54). In summary, scRNA-seq studies offer an unbiased approach for identifying both known ciliary genes and potential ciliary candidates, along with genes specific to motile and primary cilia.

### Comparative genomics (phylogenetic profiling) reveals previously unidentified ciliary candidate genes

**Figure 4.**
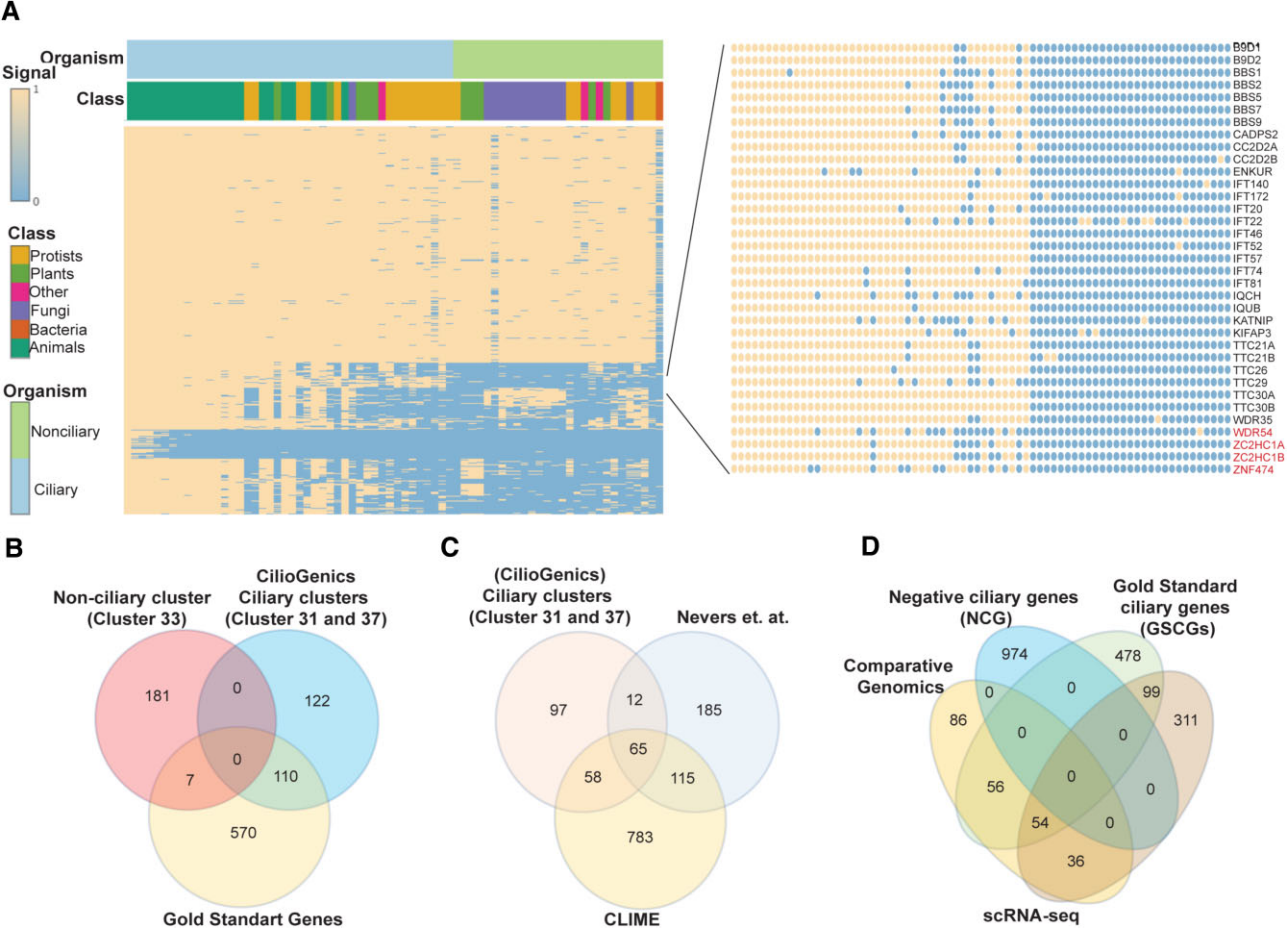
Comparative genomics (phylogenetic profiling) identifies previously unknown ciliary candidate genes. (**A**) The heatmap depicts the representative of the clusters, including non-cilia-specific clusters and cilia-specific clusters. The heatmaps of genes in a cilia cluster (cluster 31) are shown. The candidate genes, such as *WDR54, ZNF474*, and *ZC2H1A*, are depicted with a red font, while the known ciliary genes are distinguished with a black font. (**B, C**) The Venn diagram illustrates the comparison between the gene lists from a non-cilia-specific cluster (cluster 33) and cilia-specific clusters (clusters 31 and 37). The diagram specifically compares the ciliary candidate genes identified in the gene list from CilioGenics (this study and cluster 31 and 37) with those from CLIME (Clustering by Inferred Models of Evolution, https://gene-clime.org/) and the work by Never et al. (68,69). (**D**) The Venn diagram depicts the comparison of gene lists from Negative Ciliary Genes (NCGs) and Gold Standard Ciliary Genes (GSCGs), along with cilia-specific clusters (cluster 31 and 37), and the single-cell RNA sequencing (scRNA-seq) data derived from this study (CilioGenics).

Because not all eukaryotic species need the specialized function of cilia, cilia have been lost in many organisms, including plants and fungi. The assumption is that genes with a unique role for cilia should be present in the genomes of ciliated organisms but not in the genomes of organisms without cilia. For this reason, comparative genomics has been extensively employed to uncover ciliary genes. We performed comparative genomics on 72 eukaryotic species for over 20 000 human protein-coding genes to predict the human genes involved in ciliary functions and then compared the candidate ciliary genes to the candidate ciliary genes from our scRNA-seq analyses. Our comparative genomics followed by the dissimilarity matrix hierarchical clustering reveals a total of 40 gene clusters, including two cilia-specific clusters (31 )and (37), average conservation clusters like vertebrate-specific clusters (1, 4, 6, 9, 16, and 30), and non-cilia-specific clusters (low specificity clusters: 2, 3, 5, 7, 8, 10, 11, 12, 13, 14, 15, 17, 18, 19, 20, 21, 22, 23, 24, 25, 26, 27, 28, 29, 31, 32, 33, 34, 35, 36, 37, 38, 39 and 40) (Figure 4A–D). The average conservation clusters are believed to have potential ciliary genes, but their ciliary potential is lower than that of two cilia-specific clusters. This is because certain genes may lack counterparts in lower ciliary animals but have them in higher ciliary species. Visit https://ciliogenics.com to view each gene and heatmaps for clusters. With a combined total of 232 genes, the cilia-specific clusters 31 and 37 contain 159 and 73 putative ciliary candidate genes, respectively. To assess the predictability of the cilia-specific clusters (31) and (37) and figure out the randomness of predictions, we compared the gene lists of a non-ciliary cluster (cluster 33, which contains 188 genes) and the cilia-specific clusters (31) and (37) against the gene list from Gold Standard Ciliary Genes (GSCGs). The Venn diagram illustrated that nearly half of the genes in the ciliary clusters (31) and (37) overlapped with the GSCGs, whereas only 3.7% of the genes in cluster 33 were part of the GSCGs (Figure 4B). Additionally, we compared the gene list from the cilia-specific cluster with the gene list from previous comparative genomics studies (68,69) (Figure 4C). Of these 232 genes in the cilia-specific cluster, 110 are known ciliary genes, while the rest are putative ciliary genes, including 36 genes shared by our four human scRNA-seq analyses (Figure 4D and Supplementary Table S5). These 36 genes, including WDR54, ZC2HC1A, and ZNF474, are all likely to be strong ciliary gene candidates because they were found independently by two different approaches. Surprisingly, 311 genes were discovered solely through four human scRNA-seq analyses, whereas only 86 genes were revealed through comparative genomics analysis, suggesting the independent potential of each method for revealing novel ciliary candidate genes (Figure 4D).

### Gene regulatory network and protein-protein interactions (PPI)

Transcription factors and cilia-specific motifs have been extensively used to reveal ciliary candidate genes. Here, we next dig into the transcription factors that are known to regulate them. Chen et al. used scRNA-seq data to identify genetic regulatory networks using nine different species for the lung, and this approach revealed transcription factor (TF)-target interactions (70). Here, we have used the transcription factor (TF)-target interactions for ciliated cells, in combination with the scRNA-seq and comparative genomics dataset described above, to identify candidate ciliary genes. Specifically, we chose six cilia-related transcription factors (FOXJ1, RFX2, RFX3, JAZF1, GLIS3 and MYB). JAZF1 has emerged as a ‘regulator of cilia differentiation’ and is thought to operate upstream of FOXJ1 (an F-box transcription factor) expression, which is required for constructing motile cilia (71–76). We reasoned that if FOXJ1 functions downstream of JAZF1, the expression of ciliary genes should be influenced by both; thus, we include both in our study (73,76). GLIS3 was found to localize to the primary cilium and is required for renal cilium formation, which suggests that it might regulate cilia-related gene expressions (74).

**Figure 5.**
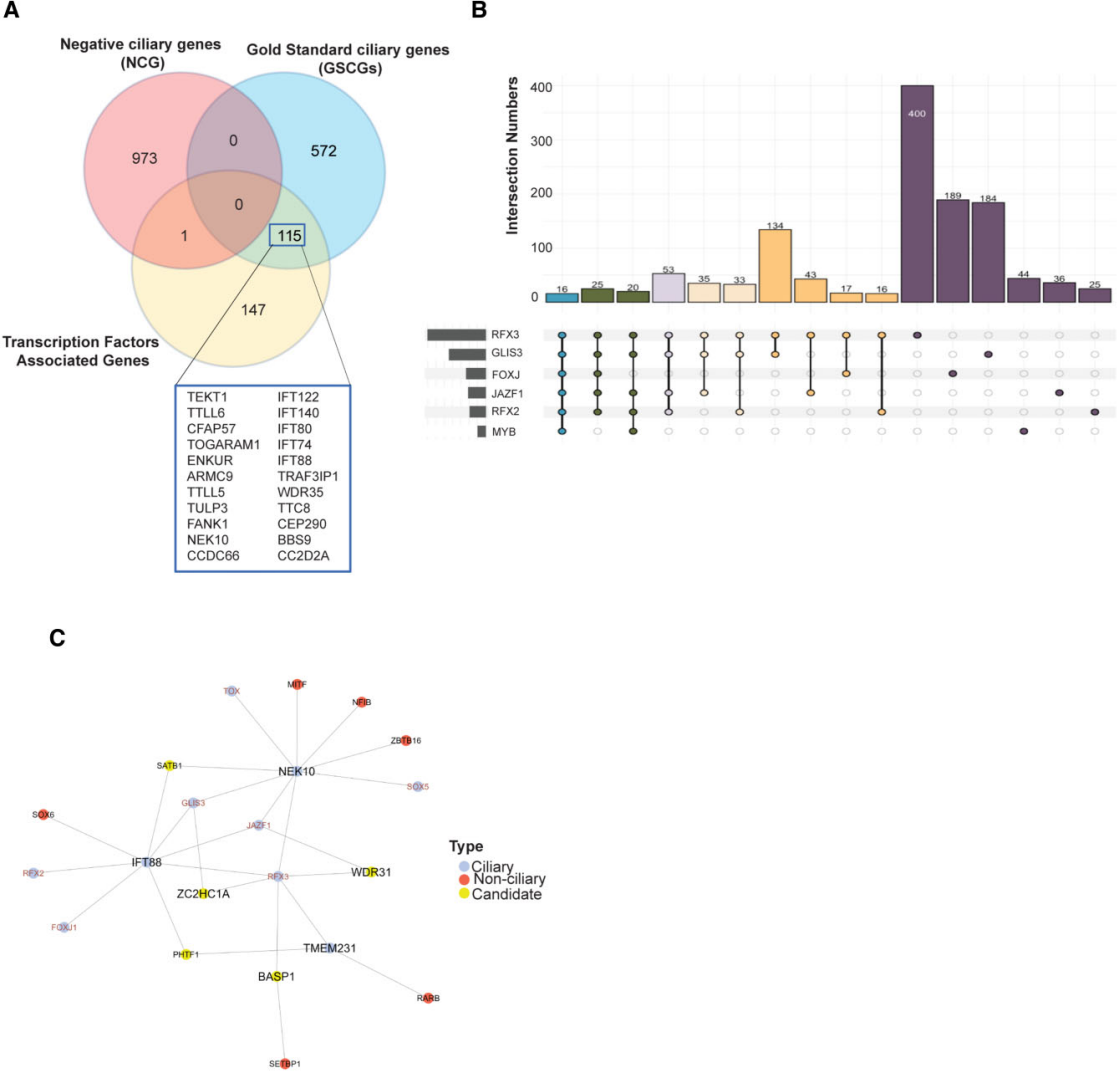
Transcription factor (TF) analysis reveals many new ciliary candidate genes. (**A**) The Venn diagram depicts the comparison of gene lists from Negative Ciliary Genes (NCGs) and Gold Standard Ciliary Genes (GSCGs), along with the binding targets of ciliary TFs (FOXJ1, RFX2, RFX3, MYB, GLIS3 and JAZF1) derived from this study (CilioGenics). For representation purposes, 263 distinct genes regulated by three different ciliary TFs were included in the comparison. (**B**) The number of intersections of FOXJ1, RFX2, RFX3, MYB, GLIS3 and JAZF1 target genes is shown in the Upset plot. Sharing of TFs, including singles, doubles, triples, quartets, quintuples, and sixes are shown in different colors. (**C**) Network analysis shows the binding targets of ciliary TF. Ciliary genes, candidate genes, and non-ciliary genes are shown in blue, red, and yellow, respectively. The TFs are labeled with red font color. Lines display regulatory interactions between TFs and their target genes. The network illustrates the regulation of well-known ciliary genes including *NEK10* and *IFT88* by three ciliary TFs: GLIS3, JAZF1 and RFX3. Furthermore, the ciliary candidate gene *ZC2HC1A* is regulated by two ciliary TFs, including GLIS3 and RFX3.

In our genetic regulatory network analysis (see methods for data analysis), we discovered that GLIS3 is likely involved in the regulation of 562 genes in ciliated cells, including 127 GSCGs. It is essential to highlight that we used the latest version of the GSCG list for our comprehensive comparisons. Genes not found in the updated GSCG list were considered unknown (55). JAZF1 regulates 270 cilia-related genes, including 89 GSCGs, whereas RFX2 regulates 99 Gold Standard ciliary genes among its 246 genes. RFX3 regulates 178 Gold Standard ciliary genes out of 878 regulated genes, while MYB regulates as 50 GSCGs out of 126 genes, and FOXJ1 regulates 107 GSCGs out of 433 genes (Supplementary Table S6). Further analysis revealed that at least three, and potentially all, of these distinct ciliary TFs (RFX2, RFX3, MYB, GLIS3, JAZF1 and FOXJ) regulate 263 genes. The complete list of genes and associated TFs can be found in Supplementary Table S6. To ensure high confidence in target genes regulated by these ciliary transcription factors (TFs), we focused on genes regulated by at least three TFs. Among these 263 genes, 115 are GSCGs (Figure 5A). Additionally, six ciliary TFs exert influence on 16 genes, including ciliary candidate genes (SPAG17, STK33, CCDC170, ERICH3, CCDC146, and CCDC113) and GSCGs (AK7, ARMC2, ULK4, SYNE1, FANK1, CFAP43 and CCDC39) (Figure 5B and Supplementary Table S6). Not surprisingly, CCDC170, ERICH3, CCDC146 and SPAG17 were found to localize to cilia or the base of cilia, suggesting that our TF-based network can uncover novel and unknown ciliary genes (Figure 5B, C and Supplementary Table S6)) (40,51).

**Figure 6.**
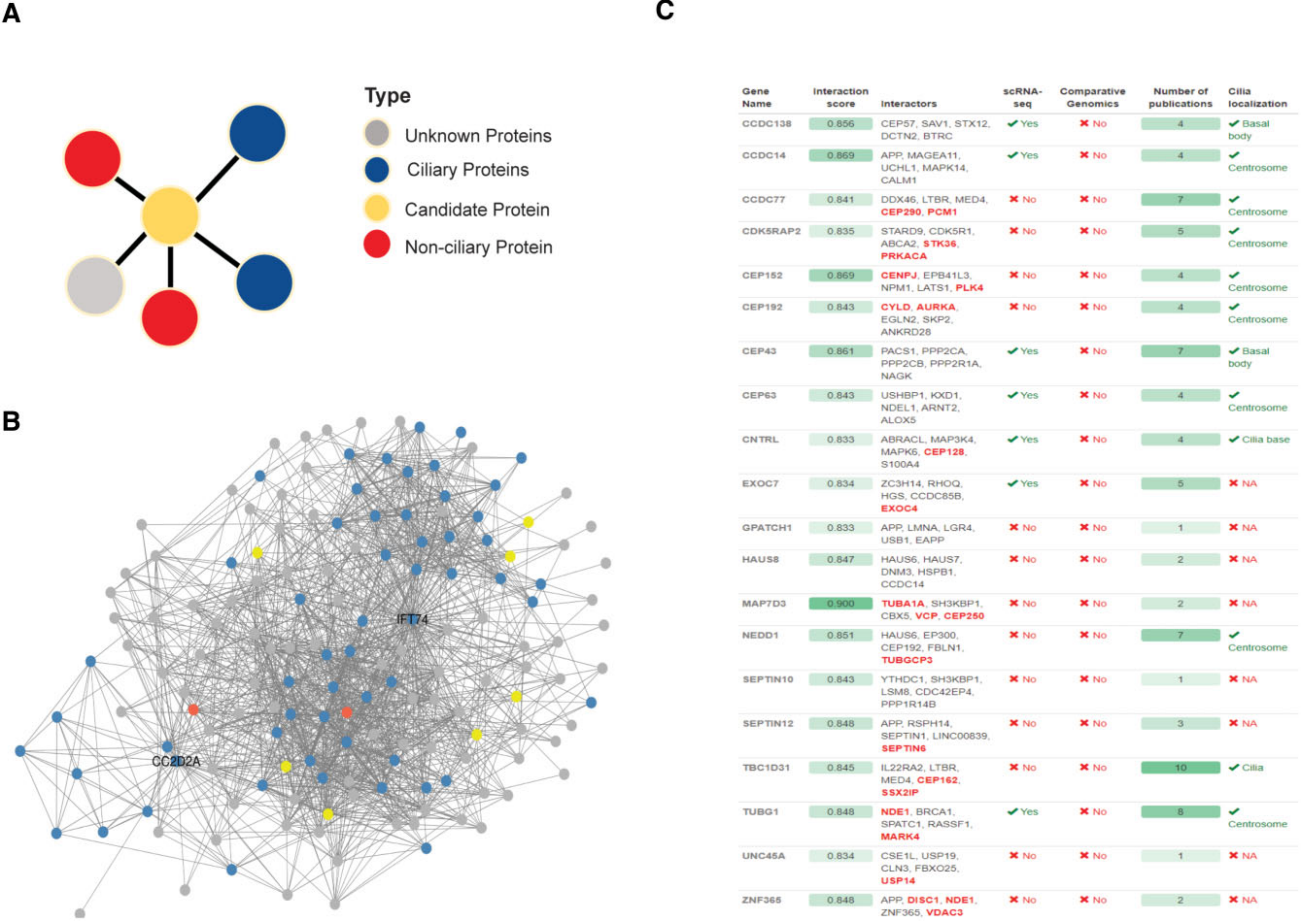
PPIs identify novel ciliary candidate genes. (**A**) Shown is the procedure for identifying ciliary genes using Protein-Protein Interactions (PPI). Each human protein is categorized as a ciliary, negative, ciliary candidate, or unknown, with distinct color labels. The scoring mechanism involves assigning positive or negative points to each human protein based on their interactions. Gold standard ciliary genes (GSCGs): When a candidate protein interacts with a protein from the GSCGs, it receives a positive score. Non-Ciliary Proteins (NCGs): No negative scores are assigned when a candidate protein interacts with a protein classified as non-ciliary (Negative Genes). Unknown Status: Proteins with unknown ciliary status are also considered in the analysis. Additionally, the number of interactions with known ciliary proteins is also used to evaluate the power of the PPI network for specific candidate proteins. (**B**) PPIs of IFT74 and CC2D2A are shown in the network. The red, blue, green, and yellow denote ciliary, negative, unknown, and ciliary candidate genes, respectively. (**C**) Table displays the top genes from PPIs. The table shows interaction scores, interaction partners, ciliary candidacy from scRNA-seq and comparative genomics, the number of publications found, and cilia localization statutes.

Next, we downloaded all publicly available protein-protein interactions (PPI) data from IntAct, BioGRID and HuRI (77–79). We hypothesized that the PPIs could reveal novel ciliary genes as proteins that function in the same organelle and/or cellular regions, like cilia, will most likely display intracellular physical interactions (Figure 6A). We first categorized the proteins into 3 groups: (i) known ciliary components (i.e. in the GSCGs); (ii) non-ciliary proteins (i.e. in the NGS datasets published); and (iii) ‘unknown’ (containing possible ciliary proteins) (68,80). When a candidate protein interacts with a protein from GSCGs, it receives a positive score, but no negative scores are assigned when it interacts with a protein classified as non-ciliary. Additionally, we took into consideration the strength of the PPI network for individual candidate proteins in terms of how many known ciliary proteins are present (Figure 6A). Notably, sub-networks like IFT74 and CC2D2A contain numerous known ciliary proteins (Figure 6B). Focusing on three candidate ciliary proteins (WDR54, ZC2HC1A and ZNF474) identified in the single-cell RNA sequencing and comparative genomics analyses mentioned earlier, it revealed that ZC2HC1A interacts with known ciliary proteins, while the other two proteins do not exhibit such interactions. Notably, CCDC138, CCDC14 and CCDC77 emerge as top candidate ciliary proteins from the PPI analysis and have indeed been previously shown to localize to the basal body (Figure 6C and Supplementary Table S7) (81–83).

Finally, we downloaded the publicly available Human Protein Atlas (also referred to as text mining) database (https://www.proteinatlas.org/), a rich source of subcellular localization data for most human proteins (84). Our automated analysis, complemented by manual inspection using the Human Protein Atlas, revealed that out of 370 proteins localizing to cilia, 75 are categorized as ‘known’ ciliary proteins, leaving the remaining 295 proteins, including BASP1, as poorly characterized novel ciliary proteins (Supplementary Table S8). Taken together, our TF network, PPI analysis and text mining uncover novel ciliary genes.

### Combined method CilioGenics is superior to any single method

**Figure 7.**
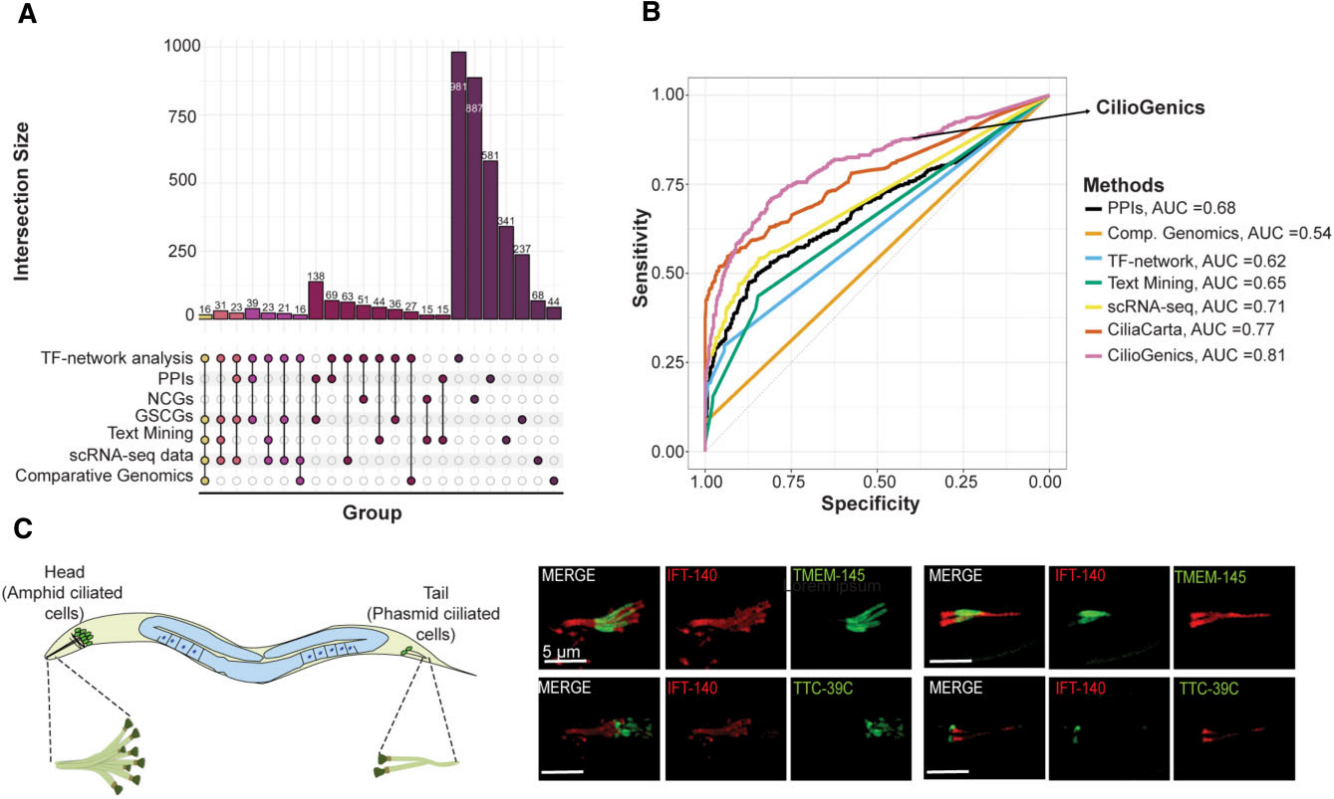
Integrated method CilioGenics is superior to any single method. (**A**) The UpsetPlot provides a comprehensive overview of the intersections and unique sets among different gene lists derived from various approaches, including scRNA-seq, PPIs, comparative genomics, TF-network analysis, and text mining. Additionally, it illustrates the overlap between Gold Standard Ciliary Genes (GSCGs) and negative ciliary genes (NCGs). Each connecting line in the diagram indicates the intersection of the indicated gene lists. (**B**) Shown is the ROC curve plot displaying the sensitivity (true positive rate) on the *y*-axis and specificity (true negative rate) on the *x*-axis. CilioGenics stands out with the highest AUC score of 0.81, surpassing the AUC scores of Comparative Genomics (0.54), PPIs (0.68), TF-network (0.62), Text Mining (0.65), scRNA-seq (0.72) and CiliaCarta (0.77). (**C**) Shown are the localization of TMEM-145 and TTC-39c in the head and tails of ciliated sensory neurons (green) in *C. elegans*. IFT-140 (also known as CHE-11, red) is used as the ciliary marker. Scale bar = 5 μm.

Our analysis reveals that the ability of each separate approach to identify known ciliary genes and prospective ciliary candidate genes varies, even though they can all find new ciliary molecular components. Single methods differ in their abilities to identify known ciliary genes and predict ciliary candidate genes (Figure 7A). TTC39A/C and TMEM145, for instance, have emerged as strong candidate ciliary genes based exclusively on scRNA-seq analysis from *C. elegans* but not based on scRNA-seq analysis from humans and the other four techniques (PPI interaction, comparative genomics, TF-network analysis, and text mining). We indeed confirmed that both TTC39A/C and TMEM145 are localized to the cilia of sensory neurons in the head (amphid) and tail (phasmid) of *C. elegans* (Figure 7C). However, this makes choosing a new cilia candidate gene more complicated because of how to prioritize the cilia candidate genes. The combined method, in which all approaches are considered, might be more successful at identifying new cilia genes and weeding out false negative candidates. To obtain more potent results and outcomes that are more biologically instructive, we evaluated the data in aggregate.

Initially, the CilioGenics score is calculated for each human gene's potential to be a ciliary gene after determining a score for each gene using inputs from scRNA-seq, PPI interaction, comparative genomics, TF-network analysis and text mining. Subsequently, we used the AUC-ROC (Area under the Receiver Operating Characteristic Curve) curve to evaluate the performance of our five distinct approaches, CilioGenics, and CiliaCarta. CiliaCarta is a database that provides a comprehensive list of genes associated with cilia, including many ciliary candidate genes (54). The ROC curve offers insights into the efficacy of the method, where a graphical representation of the trade-off between sensitivity (true positive rate) and specificity (true negative rate) is displayed. The area under the curve (AUC) displays the strength of the prediction approach. Thus, ROC curves were generated for each method, including CilioGenics, CiliaCarta, and other single methods. We need to note here that, for consistency, the same set of genes from the manually collected ciliary genes, which were not from GSCGs, were used in each analysis. Genes from this list were labeled as positive, whilst NCGs were classified as negative. Sensitivity (TPR) is measured for each approach as the ratio of positive-predicted positive genes to all positive genes, and specificity is determined as the ratio of positive-predicted negative genes to all negative genes. The change of these values with varying thresholds is plotted as the ROC curve (Figure 7B).

In our comparative analysis, scRNA-seq analysis achieved the highest AUC score of 0.72, followed by PPI analysis with 0.68. Notably, the comparative genomics approach exhibited the lowest AUC score of 0.54 among all other approaches (Figure 7B). Furthermore, in our comparative analysis, CiliaCarta exhibited an AUC of 0.77, while CilioGenics demonstrated a superior AUC of 0.81. Given that a higher AUC signifies enhanced performance, our results indicate that CilioGenics is superior to CiliaCarta and any single method in terms of its discriminative capacity when predicting ciliary genes.

### WDR54, ZNF474 and ZC2HC1A are novel ciliary genes

**Figure 8.**
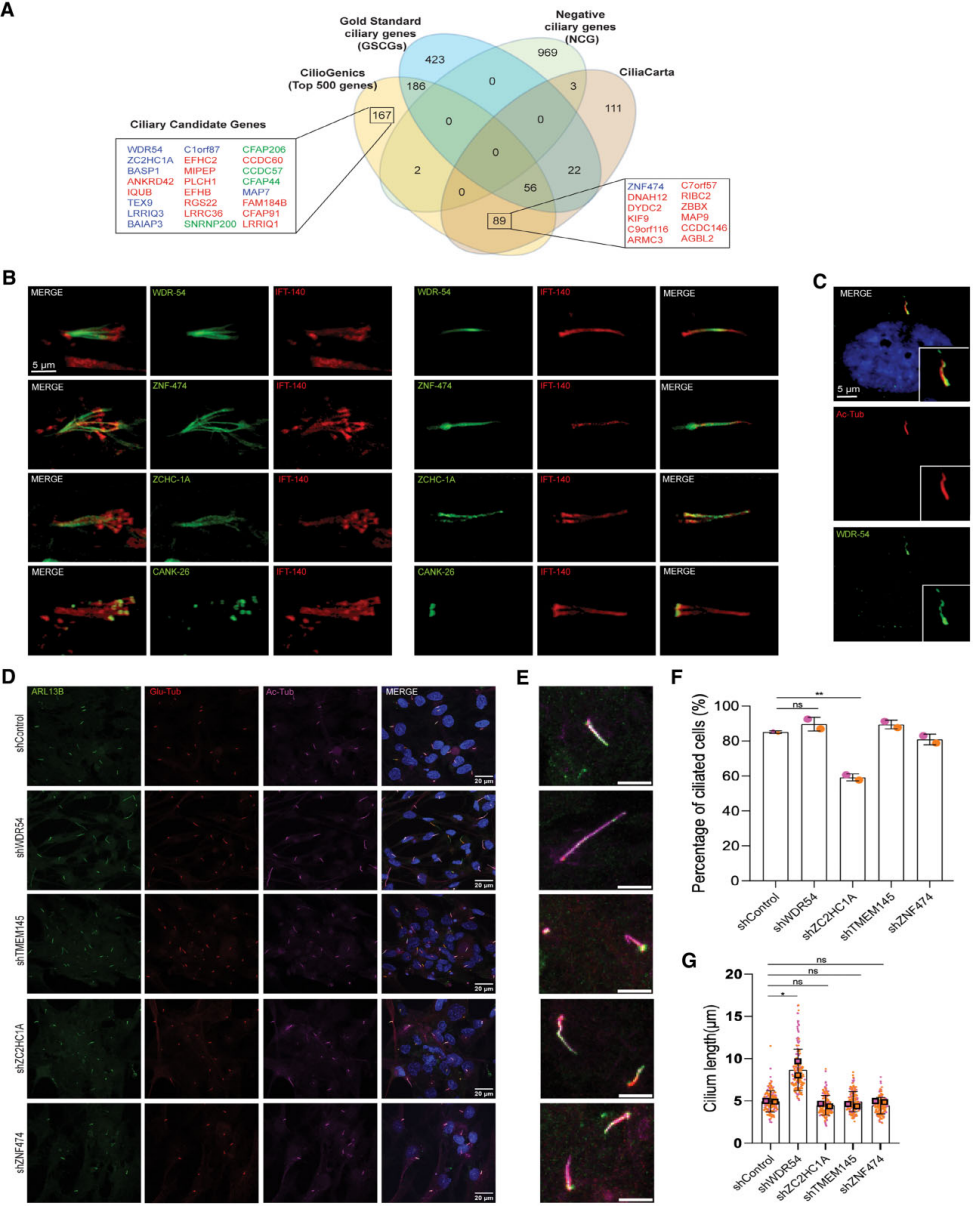
WDR54, ZC2HC1A, and ZNF474 localize to cilia. (**A**) The Venn diagram compares the top 500 CilioGenics genes to gold standard ciliary genes (GSCGs), negative ciliary genes (NCG) and CiliaCarta (54). Ciliary candidate genes, whose protein products are demonstrated to localize to cilia, are depicted in blue, those with established functions in cilia are shown in green, while the red color indicates that the functions of the gene in cilia are yet to be established. (**B**) Fluorescence images display the cilia localization of CANK-26 (the human ANKRD26 ortholog), ZCHC-1A, WDR-54 and ZNF-474 (green) in the head and tails in *C. elegans*. IFT-140 (red) marks cilia. (**C**) Localization of WDR54 (green) acetylated tubulin (Ac-Tubulin, red), and nucleus (blue) are shown in human retinal pigment epithelial-1 (RPE-1) cells. Scale bar = 5 μm. (**D**) RPE1-shRNA stable lines (control, WDR54, TMEM145, ZCHC1A, ZNF474) were fixed after 48 h serum starvation. Cells were stained with anti-Arl13b (ARL13B,green) for ciliary axonome, anti-polyglutamylated tubulin (Glu Tub, red) for basal body, anti-acetylated tubulin (Ac Tub, magenta) cilia and DAPI (blue) for nuclei. (**E**) Adjacent panel shows enlarged images of the merged RPE1-shRNA stable line. (**F**, **G**) Ciliation percentage and cilium length are plotted. Orange and pink represent values from two independent experiments. Error bars represent standard deviation. Square boxes represent mean values. (*P < 0.5, **P < 0.01, ***P < 0.001, ****P< 0.0001, ns: not significant, *n* = 2). Ciliation percentage: 1st replica 130 cells, 2nd replica 160 cell. Cilium length: 1st replica 66 cells, 2nd replica 105 cells.

The practical validation of new proposed methods is important to confirm the computationally postulated findings, but the large number of predicted genes limits the extensive experimental execution. To put our CilioGenics method to the test in terms of predicting novel ciliary genes, we focus on the top 500 genes on the CilioGenics gene list (Supplementary Figure S7). We compared the CilioGenics gene list with the lists of NCGs (975 genes), GSCGs (688 genes) and CiliaCarta (Supplementary Figure S8). Our analysis reveals that the top 500 genes contain 2 negative genes and 242 GSCGs, suggesting our CilioGenics gene list can successfully identify the Gold Standard ciliary genes. We next focused on these two negative ciliary genes (MUC4 and TUSC3) among the top 500 CilioGenics gene lists (Supplementary Table S9). Interestingly, MUC4 has already been discovered to be expressed in ciliated cells and to localize to ciliary shafts (85). We next concentrate on the remaining, previously unknown 256 ciliary candidate genes in the CilioGenics top 500 gene list (Figure 8A). Out of the 256 genes, 89 had been previously proposed as ciliary candidate genes by CiliaCarta (Figure 8A). There were already experimental validations for 28 genes, including *BASP1, ANKRD45, DNAH12, IQUB, DYDC2, TEX9, LRRIQ3, BAIAP3, C1orf87, KIF9, EFHC2, MIPEP, C9orf116, ARMC3, PLCH1, C7orf57, EFHB, RIBC2, RGS22, ZBBX, CCDC146, AGBL2, CCDC89, EFCAB1, C21orf58, MDH1B, PPIL6* and *MAP9*, further strengthening the power of our CilioGenics methods in identifying novel ciliary genes (26) (Figure 8A). Moreover, our next step involves conducting subcellular localization analyses in mammals and *C. elegans* to determine whether these ciliary candidate genes are indeed ciliary. Our selection criteria prioritize genes that received high scores from at least two independent approaches when selecting candidate genes for confirmation. Notably, the chosen genes in this process are novel candidates not previously recognized as ciliary genes. *WDR54, ZC2HC1A* and *ZNF474* have particularly stood out among the top candidate gene list: *ZC2HC1A* at position 72 (CilioGenics mean score: 3.062), *WDR54* at position 192 (CilioGenics mean score: 2.417) and *ZNF474* at position 294 (CilioGenics mean score: 2.035). Using this approach, we had already confirmed the cilia localization of WDR31 (CilioGenics position: 977 and CilioGenics mean score: 1.250) in both human RPE1 cells and *C. elegans* (58). Confocal microscopy analysis reveals that WDR54, ZC2HC1A, and ZNF474, all under the control of the *arl-13* promoter, localize to the cilia of sensory neurons of the head and tails in *C. elegans*. Moreover, we selected the 975th-ranked ANKRD26, the *C. elegans* CANK-26, for confirmation. *C. elegans* CANK-26 (the human ANKRD26 ortholog) is enriched at the basal body of cilia (Figure 8B). Furthermore, endogenous staining with an available WDR54 antibody confirms that human WDR54 is indeed in the cilia of human RPE1 cells (Figure 8C). Next, we transduced RPE1 cells with shRNAs targeting control, *WDR54, TMEM145, ZC2HC1A* and *ZNF474* to evaluate the effects of their absence on ciliogenesis and cilia length. We stained the cells for the cilium-specific protein ARL13B (green), acetylated tubulin (magenta), and the basal body marker polyglutamylated tubulin (red) (Figure 8D and E). Compared to control shRNAs, the number of ciliated cells decreased in *ZC2HC1A* deficient cells, while cilia length remained unchanged (Figure 8F and G). Interestingly, our cilia length measurement revealed that cilia were longer in *WDR54* deficient cells compared to the control, suggesting that both *ZC2HC1A* and *WDR54* regulate the cilia biogenesis (Figure 8G). Next, we generated mutants for six newly confirmed ciliary genes (*WDR54, ZNF474, ZC2HC1A, TTC39C, ANKRD26* and *TMEM145*) using CRISPR/Cas9 and compared their cilia length to that of the wild-type. The analysis revealed that none of these single mutants affected the cilia length (Supplementary Figure S9A and B). Next, we employed two behavioral assays, chemotaxis and osmotic avoidance, to assess the functional consequences of mutations. In in *C. elegans*, cilia on the ASH sensory neurons are indispensable for osmotic avoidance response, while chemotaxis relies on functional cilia on both the AWC and AWA sensory neurons. The majority of mutants displayed chemotaxis behavior indistinguishable from wild-type worms. However, *cank-26* mutants exhibited a mild chemosensory defect, suggesting a potential role for this gene in this sensory process. Consistent with the chemotaxis results, all single mutants except *cank-26* displayed wild-type behavior, remaining trapped within the 8 M glycerol ring, which represents a high osmolarity environment. Notably, approximately 20% of *cank-26* mutants escaped the ring, indicating a partial impairment in their osmotic avoidance response (Supplementary Figure S9C and D).

## Discussion

More than 50 different papers contributed to the list of ciliary candidate genes, but even with their unquestionable contribution to the discovery of many ciliary genes, the complete list of ciliary genes is far from complete. The SYSCILIA Gold Standard (SCGSv2) publication provided a list of 687 known ciliary genes.

Consistent with this foresight, while the 2019 CiliaCarta work suggested there are 956 prospective ciliary genes, a 2021 updated SYSCILIA Gold Standard (SCGSv2) publication provided the list of 687 known ciliary genes (8–56). Many of these 57 independent works each used a different approach to find ciliary candidate genes, but their capacity to identify the same set of genes differs from one another (Supplementary Table S10). A comparison of the published lists of ciliary candidate genes reveals the names of the human genes identified by each paper, which allows us to rank genes based on the identification of several works. 12 268 different genes were picked up as ciliary candidate genes by at least one study, whereas 5782 and 2772 genes were chosen as ciliary candidate genes by only one and two studies, respectively. The gene ranking reveals that the first 44 genes are identified as ciliary candidate genes by only 20 publications; the other 37 studies failed to do so. This statistic alone shows how differently each work can disclose the ciliary candidate genes, but it also proves it has been problematic for all researchers and clinical geneticists to prioritize the ciliary candidate genes whenever they look for the genes that cause disease. Furthermore, the independent gene list produced by our five separate approaches is consistent with the aforementioned conclusion; each has a distinctive ability to discover both known ciliary genes and ciliary candidate genes. A strategy incorporating many approaches would likely perform better than each approach used alone.

Consistent with expectations, for identifying known ciliary genes and predicting novel ciliary genes, our novel combined method (CilioGenics) performs better than all other methods evaluated and CiliaCarta. In the current study, building CilioGenics scores for the ciliary potential of each human gene takes into account five different methods, including (i) PPI scores obtained by analyzing and merging PPI data from IntAct, BioGRID, and HuRI; (ii) scores for scRNA-seq data, which analyze fours scRNA-seq data from human lungs and *C. elegans*; (iii) scores for comparative genomics, which make a comparison of 72 ciliary and non-ciliary organisms; (iv) scores from TF-network analysis, which use the binding targets of FOXJ1, RFX2, RFX3, MYB, GLIS3, and JAZF1 in ciliated cells and (v) text mining scores, which come from the Human Protein Atlas. CiliaCarta integrated multiple datasets to predict the ciliary candidate genes (54). Our primary contribution to CilioGenics compared to the previously available methodologies and CiliaCarta is the integration of more diverse datasets, such as multiple scRNA-seq, comparative genomics, the Human Protein Atlas, and different sets of TFs. We demonstrated that the integrated approach is superior for predicting potential ciliary candidate genes and eliminating false-positive candidate genes.

Our comparative genomics analysis reveals that *WDR54*, *ZNF474* and *ZC2HC1A* show up as ciliary candidate genes, but none of the previous comparative genomics analyses suggested them as ciliary candidate genes. The failure of other studies to recognize them as ciliary gene candidates may have been partially explained by organism choices, the use of different thresholds, and analysis types (10,68,69). Nonetheless, these three genes showed up in our scRNA-seq study as ciliary candidate genes, which increases our confidence that they are ciliary genes. Our combined CilioGenics technique placed them among the top ciliary candidate genes; therefore, we gave them precedence and verified their cilia localizations.

Can CilioGenics accurately predict the total number of ciliary genes? This would be a challenging question to answer and depends on where the threshold will be cut. Furthermore, the current version of CilioGenics has some shortcomings in predicting all ciliary genes. First, PPIs of many human proteins are poorly characterized, and they are not well represented in the CilioGenics scoring. In addition, many of the antibodies utilized for subcellular localization studies of poorly characterized human proteins were far from ideal, and future improvements in antibody technology will be helpful in this area. Another limiting factor is that the majority of scRNA-seq data used for CilioGenics comes from motile ciliated human tissues, such as the lung and trachea, but tissues with motile cilia may not express primary cilia or tissue-specific genes. While efforts were made to extend the analysis to scRNA-seq data from tissues with primary cilia, such as the liver, pancreas, and retina, the analysis did not yield a cilia-specific cluster. Nevertheless, we generated a list of ciliary candidate genes specific to the motile cilia utilizing this scRNA-seq analysis of motile ciliated human tissue. Furthermore, we compiled a ciliary candidate gene list for primary cilia from two independent scRNA-seq datasets from sensory neurons in *C. elegans*. *C. elegans* has neuronal tissues with primary cilia. We believe a more diverse set of tissue-specific scRNA-seq data will help predictions derived from scRNA-seq datasets. Our future work will be directed at this aspect of this study to include a broader range of tissues.

While recognizing the limitations of the independent approaches, including scRNA-seqs, antibody technology, and PPIs in CilioGenics scoring, we remain confident that the number of genes in human cilia surpasses the current estimate of 688 from GSCGs. The support for this notion comes from the localization of 295 previously uncharacterized proteins in the Human Protein Atlas, all specific to cilia, thereby increasing the total number of identified ciliary genes to 982. Furthermore, the current study also validates the localization of five additional ciliary genes. Indeed, our work identifies *WDR54* and *ZC2HC1A* as novel regulators of cilia biogenesis. Depletion of *WDR54* leads to elongated cilia, whereas *ZC2HC1A* deficiency results in reduced cilia number. Further work is necessary to elucidate the precise mechanisms by which these proteins regulate cilia biogenesis.

In summary, we believe that CilioGenics in conjunction with our up-to-date CilioGenics website (https://ciliogenics.com/), would be helpful to scientists in searching for ciliary candidate genes, and they could use the provided dataset to prioritize their candidate genes. Additionally, users can find the number of publications where a gene was proposed as a ciliary candidate gene, check the presence of the gene in different types of comparative genomic clusters (cilia organism-specific clusters, average conservation clusters, or low-specific clusters), or examine expression patterns of genes in the human lungs. The current version of CilioGenics and the website will be openly updated, and other data sets, including scRNA-seq of other tissues, will be integrated.

## Supplementary Material

gkae554_Supplemental_Files

## Data Availability

The CilioGenics database is available at https://ciliogenics.com/. The data underlying this article are available in Zenodo at https://zenodo.org/doi/10.5281/zenodo.11526801.
