# Supplementary material for: CilioGenics: an integrated method and database for predicting novel ciliary genes
Source: Nucleic Acids Res. 2024 Jul 11;52(14):8127–45. doi: 10.1093/nar/gkae554 (PMC11317154; doi:10.1093/nar/gkae554)
Supplement: gkae554_Supplemental_Files [file gkae554_supplemental_files.zip › Supplementary Figures.pdf]

Supplementary Files:

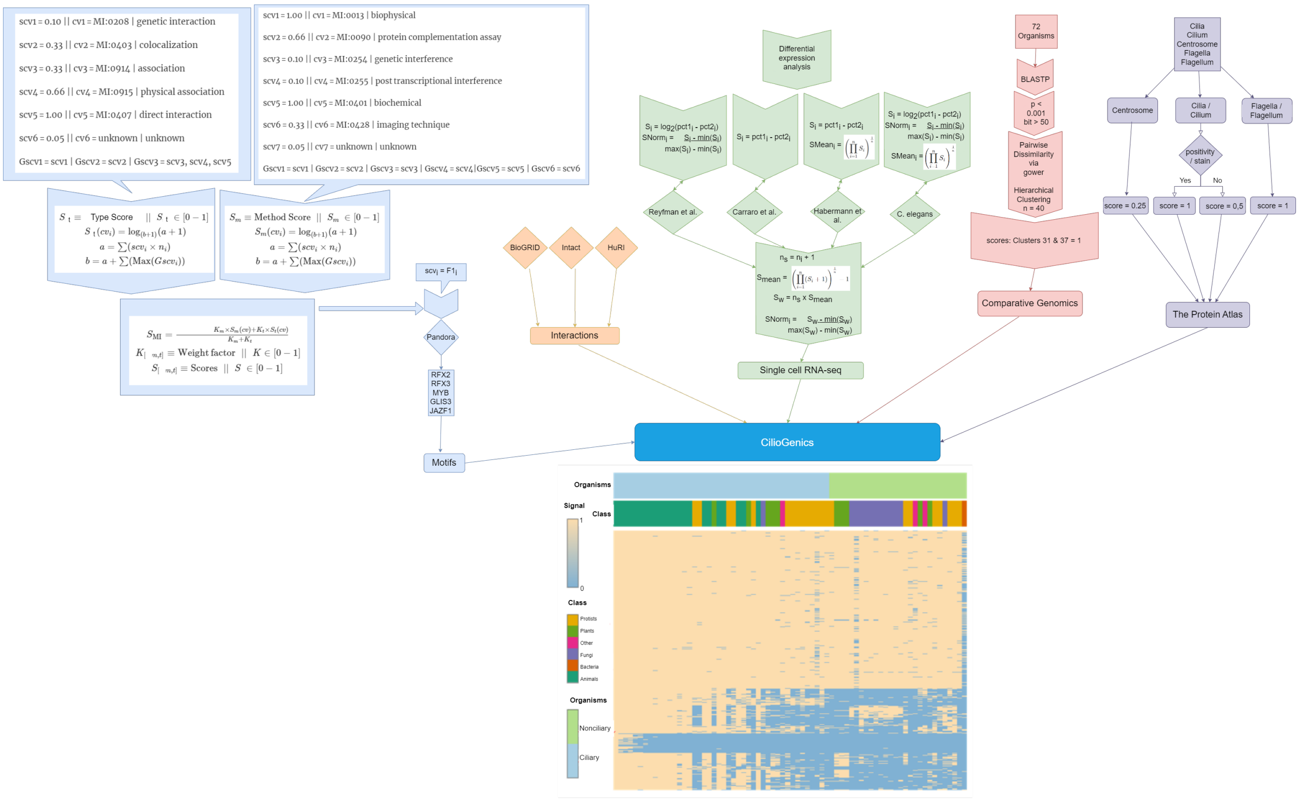

Supplementary Figure 1

The workflow of the Ciliogenics methods and scoring is illustrated.

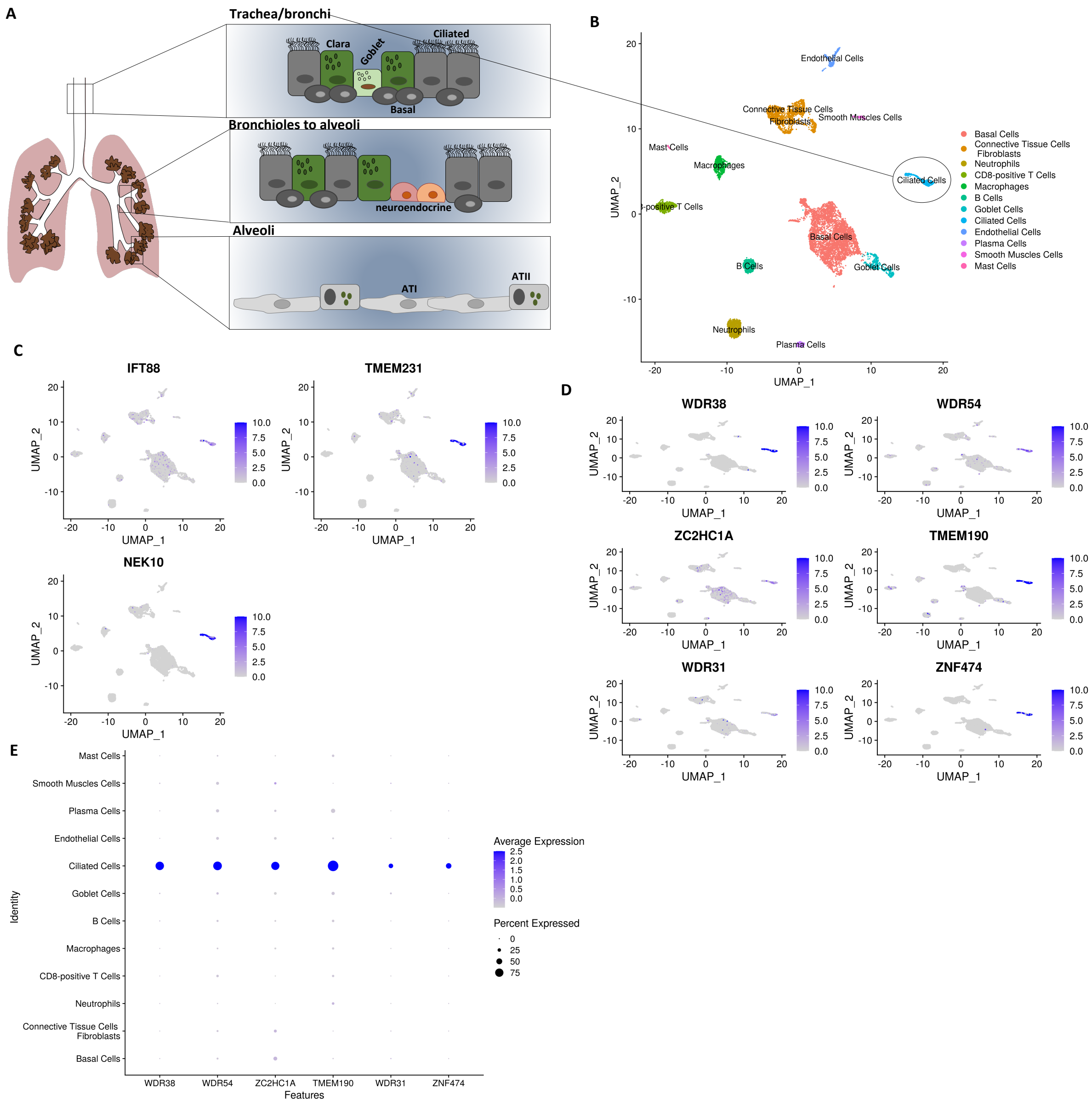

Supplementary Figure 2. Trachea

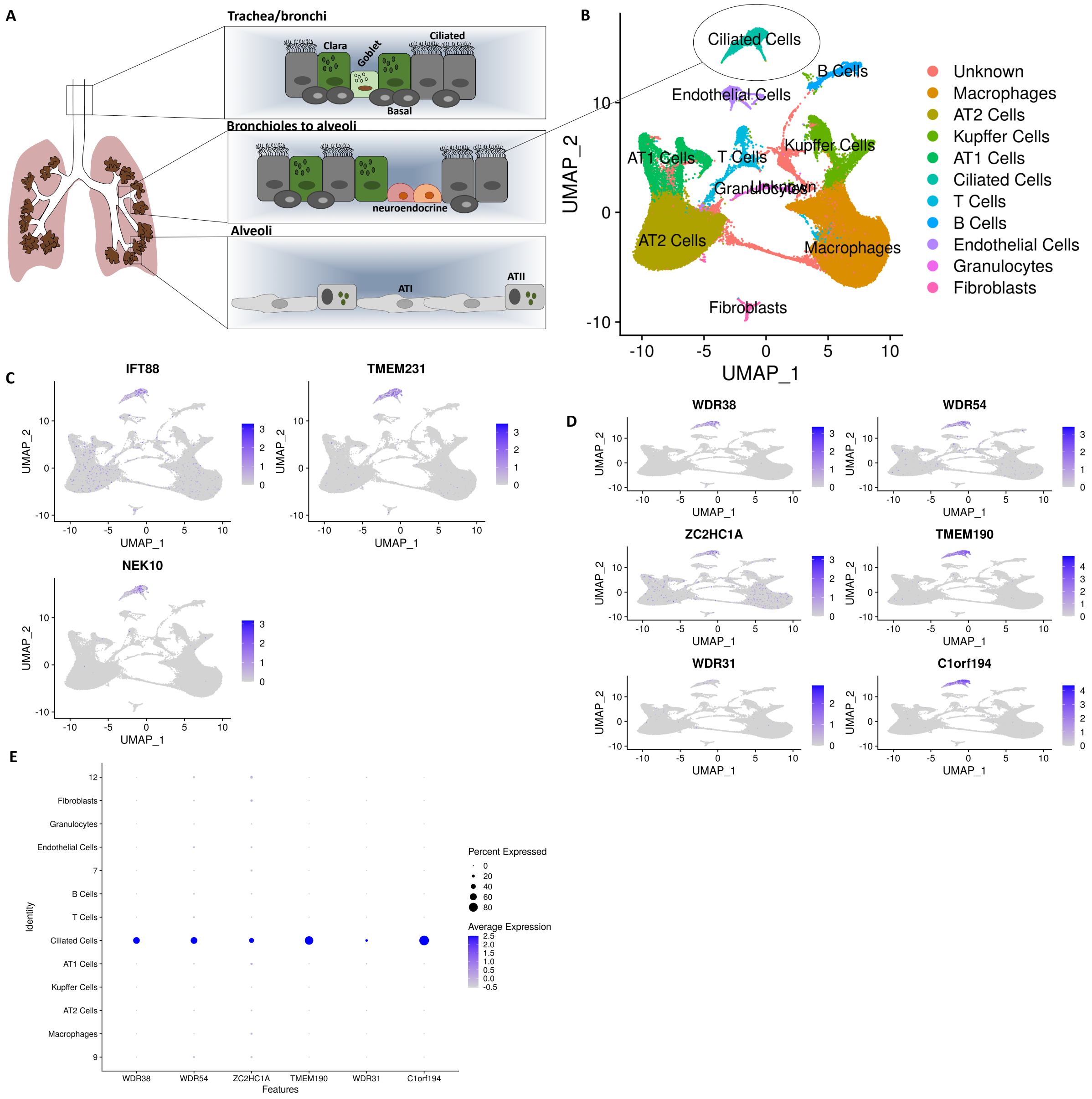

Supplementary Figure 3. Lung, Reyfman

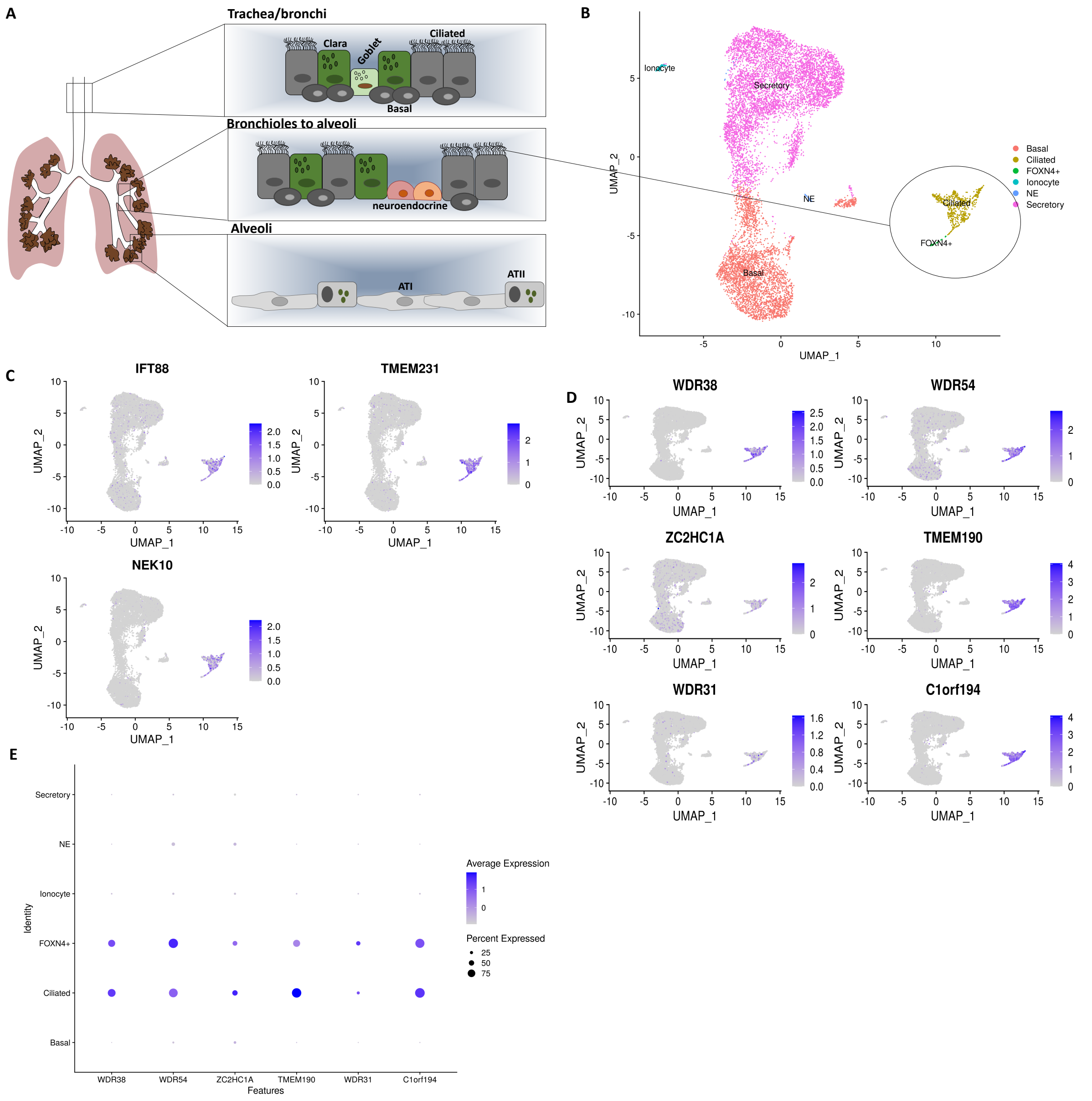

Supplementary Figure 4. Lung, Carraro

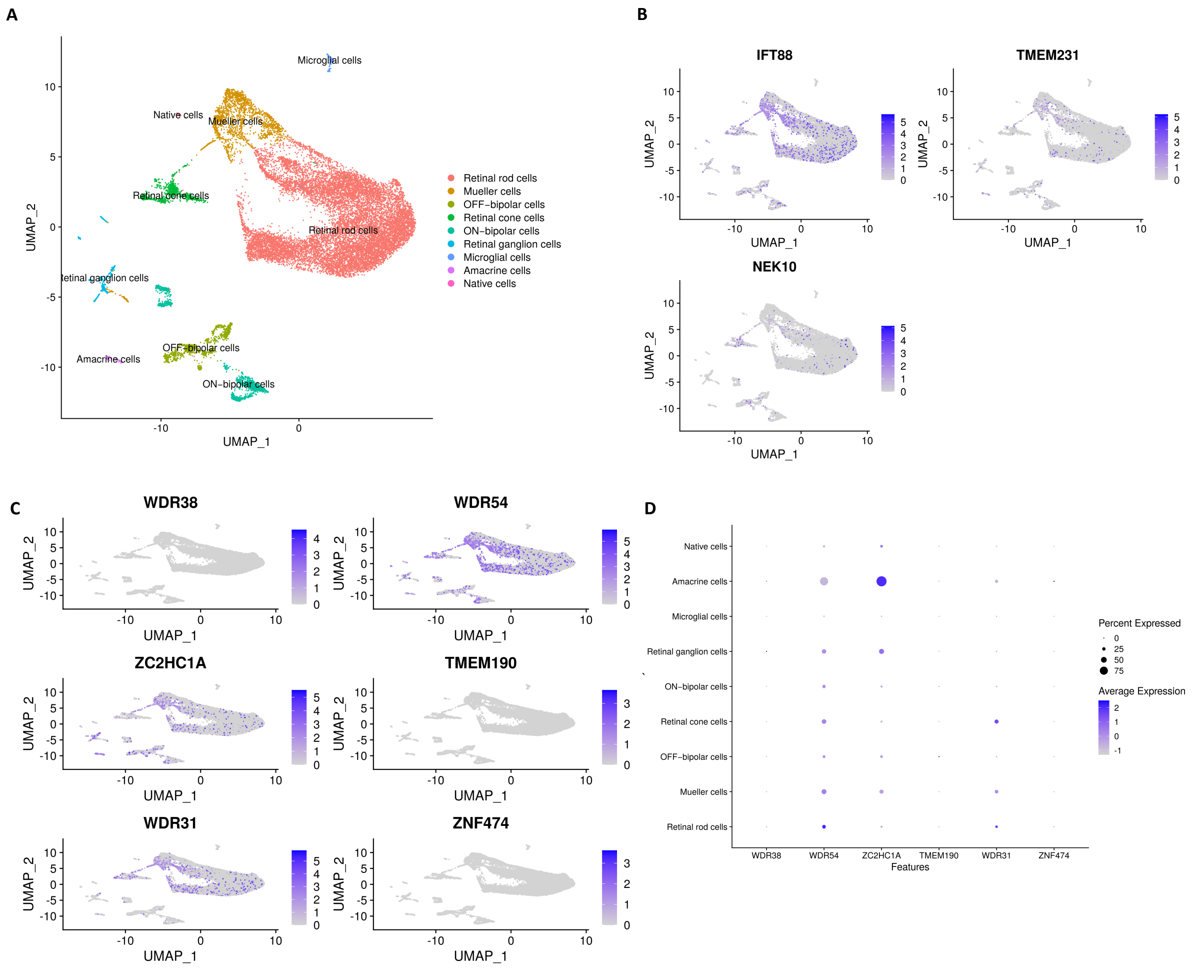

Supplementary Figure 5. Brain

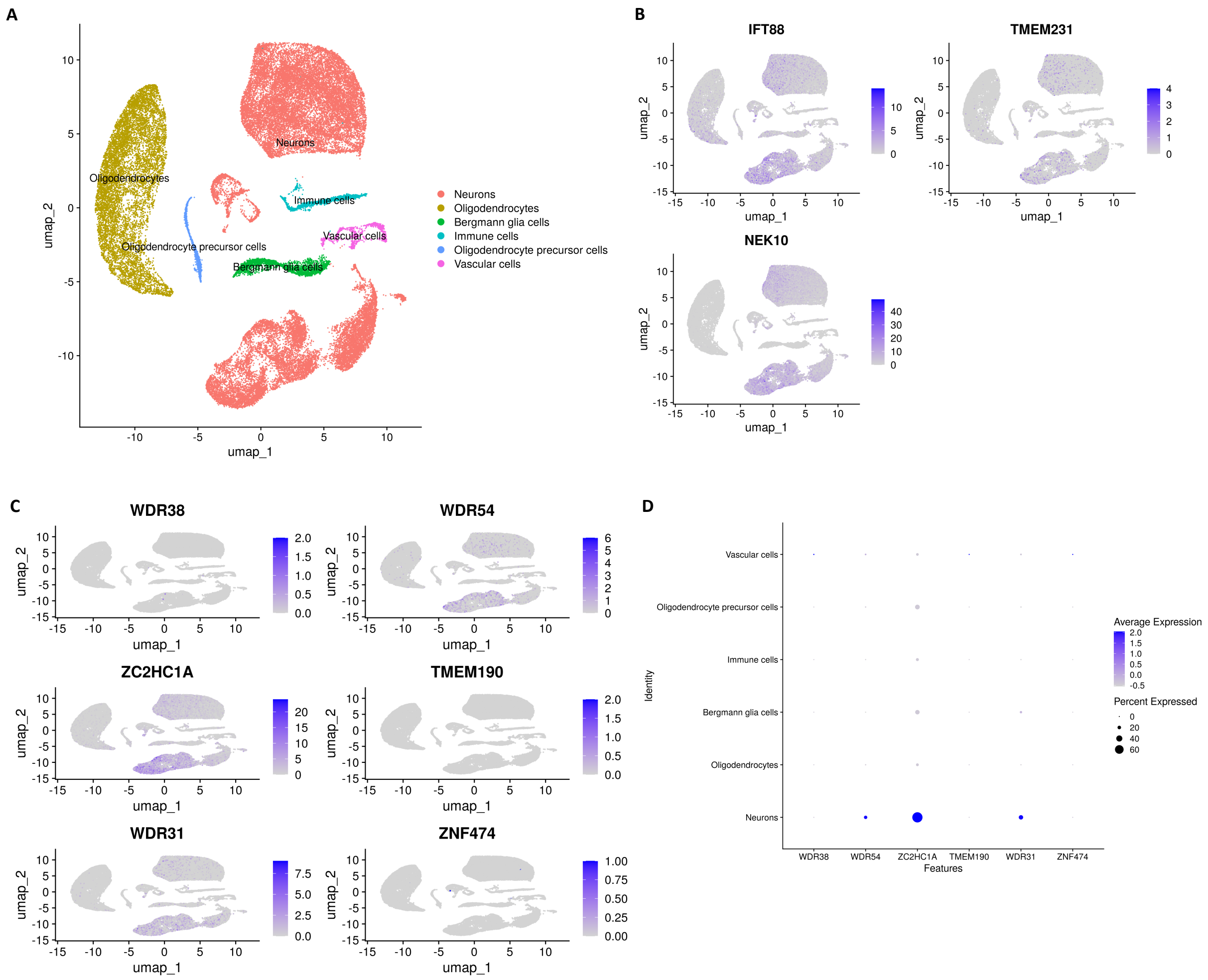

Supplementary Figure 6. Retina

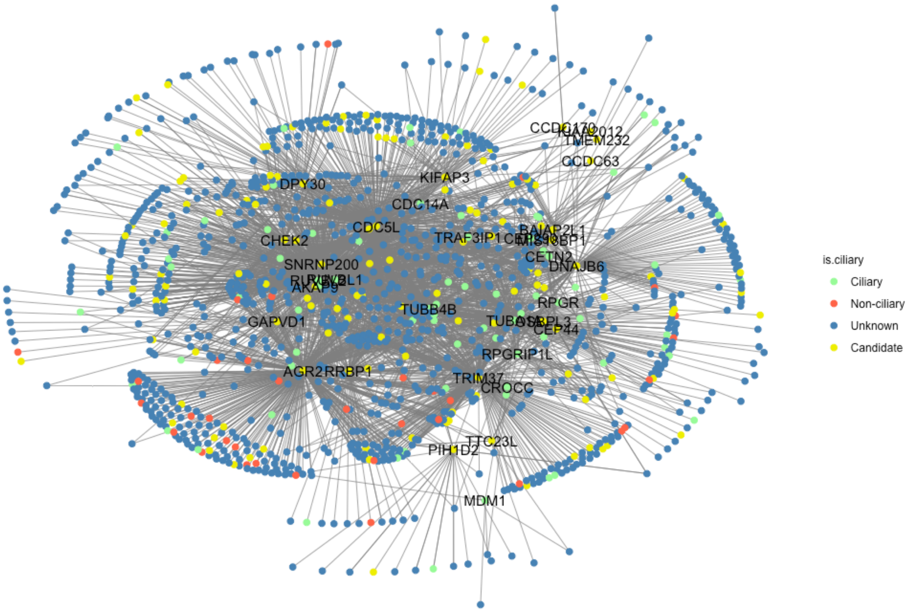

Supplementary Figure 7

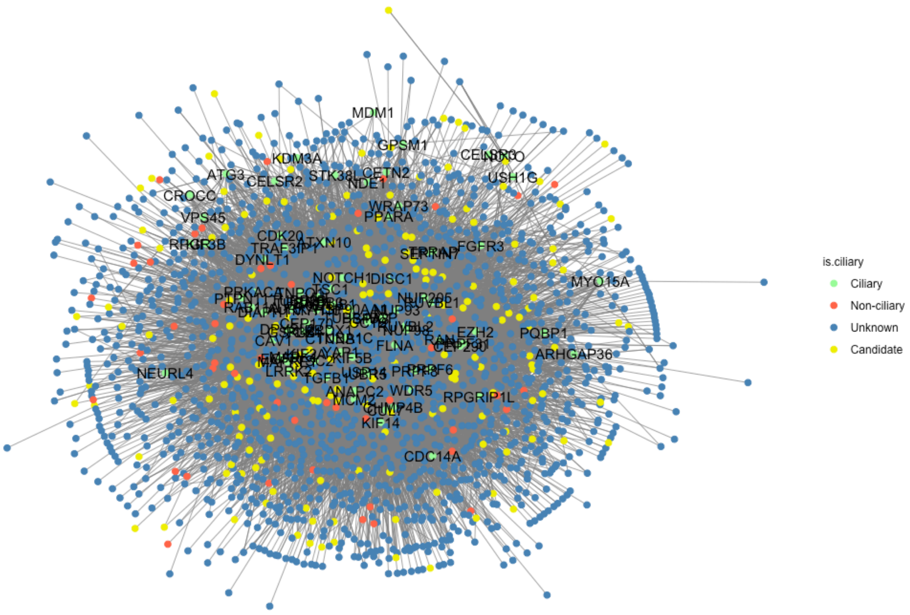

Supplementary Figure 8

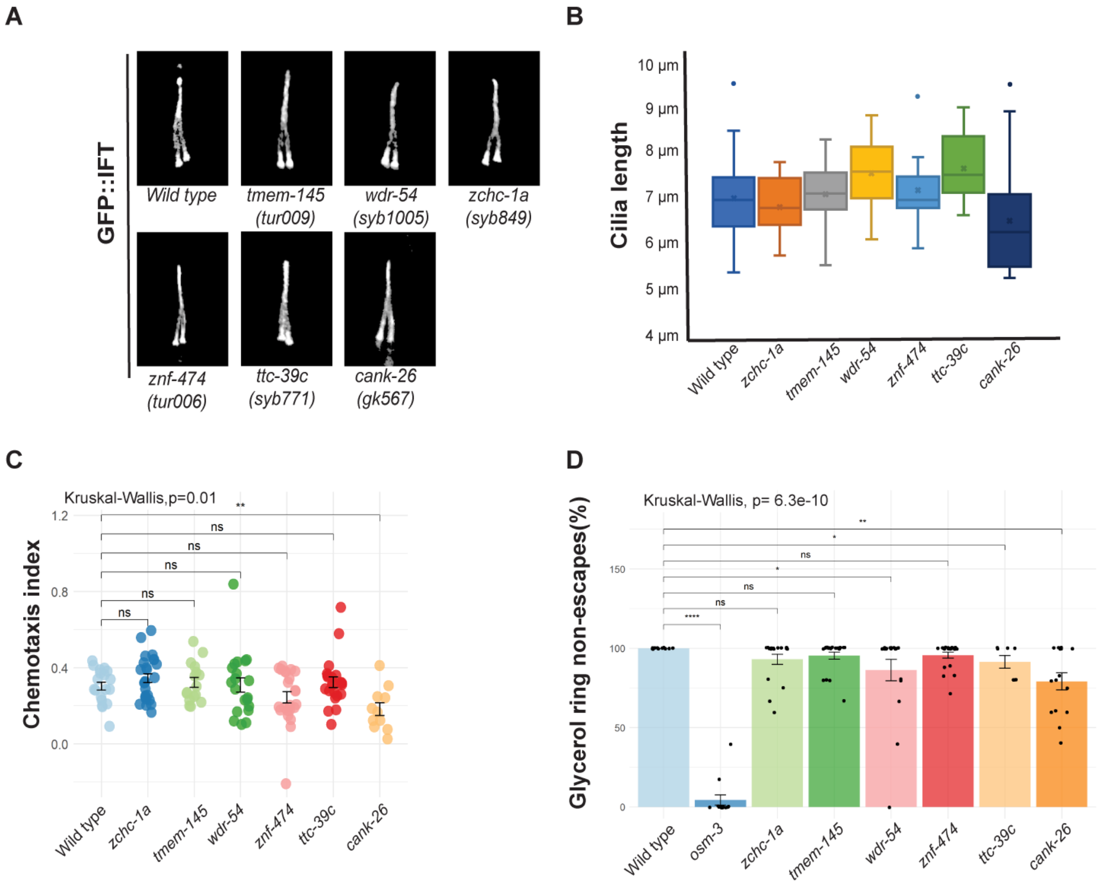

Supplementary Figure 9

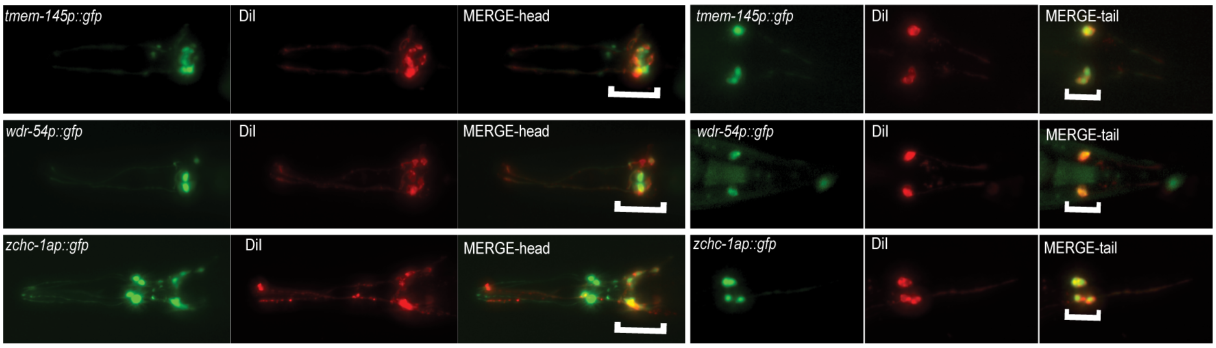

Supplementary Figure 10

Supplementary Tables

Supplementary Table 1:

The list of ciliary candidate genes from *C. elegans* scRNA-seq.

Supplementary Table 2:

The human scRNA-seq list of ciliary candidate genes from Carraro, Reyfman, Habermann, Murthy, and Trachea. Indeed, ciliary genes are indicated. Carraro, Reyfman, Habermann, Murthy, and Trachea are compared and shown in the gene list. The number of shares and the names of the studies were provided.

Supplementary Table 3:

The Gold standard ciliary gene (GSCG) list was acquired from Vasquez et al (55).

Supplementary Table 4:

A negative gene list was obtained from Nevers et al (65)

Supplementary Table 5:

The list of ciliary candidate genes (only clusters 31 and 37) from comparative genomics. Known ciliary genes and ciliary candidate genes from scRNA-seq are labeled.

Supplementary Table 6:

A list of gene targets of six cilia-related transcription factors (TF), including RFX2, RFX3, MYB, GLIS3, JAZF1, and FOXJ1, is shown. The known ciliary genes are marked.

Supplementary Table 7:

Top candidate genes from the protein-protein interaction (PPI) data for IntAct, BioGRID, and HuRI (score >0.7). PPI's top candidate genes are compared to a list of negative and gold standards. All genes (11229 genes) from PPIs are provided.

Supplementary Table 8:

List of genes from text mining from Protein Atlas. The known ciliary genes are labeled yes.

Supplementary Table 9:

The top 500 genes identified by CilioGenics have been provided, and for the complete list along with scores, you can download the data from the following link: https://ciliogenics.com/

Supplementary Table 10:

The list includes the names of the genes as well as the number of articles that have identified them as ciliary candidate genes. The publication names are shown. Documentation on chosen articles is supplied, including publication year, file names, organism kinds, and paper webpage.

Supplementary Table 11:

The organism names used in comparative genomics are listed. Additionally, the protein sequences for each organism are retrieved from NCBI, and download links are provided.

Supplementary Table 12:

Sequences of primers used for genotyping and sgRNA sequence are shown.

Package Versions.

Package	version
geneName	0.2.2
RColorBrewer	1.1.2
tidyr	1.1.3
readxl	1.3.1
DT	0.17
monocle	2.9.0
DDRTree	0.1.5
irlba	2.3.3
VGAM	1.1.5
Matrix	1.3.3
plotly	4.9.3
ggplot2	3.3.3
bsplus	0.1.2
highcharter	0.8.2
SeuratObject	4.0.1
Seurat	4.0.1
tippy	0.1.0
ComplexHeatmap	2.6.2
circlize	0.4.12
waiter	0.2.0
dashboardthemes	1.1.3
V8	3.4.0
dplyr	1.0.6
shinyBS	0.61
data.table	1.14.0
cicerone	1.0.5.9000
iheatmapr	0.5.1
networkD3	0.4
reactable	0.2.3.9000
shinycssloaders	1.0.0
shinyWidgets	0.6.0.9000
shinyjs	2.0.0.9000
RMySQL	0.10.21
DBI	1.1.1
Homo.sapiens	1.3.1
TxDb.Hsapiens.UCSC.hg19.knownGene	3.2.2
org.Hs.eg.db	3.12.0
GO.db	3.12.1
OrganismDbi	1.32.0
GenomicFeatures	1.42.3
GenomicRanges	1.42.0
GenomeInfoDb	1.26.7
AnnotationDbi	1.52.0
IRanges	2.24.1
S4Vectors	0.28.1
Biobase	2.50.0
BiocGenerics	0.36.1
shinydashboardPlus	2.0.0
shinydashboard	0.7.1
shiny	1.6.0
