# Supplementary material for: CilioGenics: an integrated method and database for predicting novel ciliary genes
Source: Nucleic Acids Res. 2024 Jul 11;52(14):8127–45. doi: 10.1093/nar/gkae554 (PMC11317154; doi:10.1093/nar/gkae554)
Supplement: gkae554_Supplemental_Files [file gkae554_supplemental_files.zip › Supplementary Material Legends_clean.pdf]

Supplementary Files:

Supplementary Figure 1

The workflow of the Ciliogenics methods and scoring is illustrated.

Supplementary Figure 2 (trachea) Supplementary Figure 3 (Lung, Reyfman) and 4 (Lung, Carraro)

Human trachea and lung scRNA-seq reveal many ciliary candidate genes. A) Shown is an illustration of the human lung, featuring cells ranging from the trachea/bronchi and bronchioles to alveoli. Various cell types, including Clara, goblet, and multiciliated epithelial cells, are highlighted.

B) The UMAPs derived from human trachea single-cell RNA sequencing (scRNA-Seq) data are displayed.

C) UMAPs of IFT88, TMEM231, and NEK10 from human trachea scRNA-Seq are presented.

D and E) Shown are UMAPs and Dotplots illustrating the expression patterns of the indicated ciliary candidate genes in the human trachea scRNA-Seq data.

Supplementary Figure 5 (Brain) and Supplementary Figure 6 (Retina)

Figure S4 displays single-cell RNA sequencing (scRNA-seq) data from the brain (Hypophthalmus), while Figure S5 presents scRNA-seq data from the retina.

A) Shown are UMAP of scRNA-seq from the brain (Hypophthalmus) and human retina.

B and C) The UMAPs generated from scRNA-Seq data of the brain's hypothalamus region are displayed for IFT88, TMEM231, NEK10, and the selected genes.

D) The Dotplot depicts the expression patterns of the chosen ciliary candidate genes in scRNA-seq from the brain (Hypophthalmus).

Supplementary Figure 7 and Supplementary Figure 8

Networks comprising the top 500 genes from CilioGenics (**Figure S7**) and 687 genes from Gold Standard (**Figure S8**) are shown. The red, blue, green, and yellow denote ciliary, negative, unknown, and ciliary candidate genes, respectively.

Supplementary Figure 9

A) Shown are cilia marked with a green fluorescent protein (GFP)-tagged intraflagellar protein IFT-74 in strains of both wild-type and the indicated mutant backgrounds. Scale bar: 3 μm

B) Shown are the measurements of cilia lengths in the wild-type and the indicated mutant genes. At least 20 cilia were measured.

C) Shown are the chemotaxis of index in the wild type and the indicated mutant genes. At least 10 independent analyses were performed for each strain. Kruskal- Wallis was used for statistical analysis. “ns” refers to non-significant.

D) Shown are Osmotic avoidance of 8 M glycerol in the wild type (positive control), *osm-3* (negative control) and the indicated mutants. Kruskal- Wallis was used for statistical analysis. “ns” refers to non-significant.

Supplementary Figure 10

Merged GFP- fluorescence images for *tmem-145p::gfp, wdr-54p::gfp, zc2c-1ap::gfp* following a dye uptake assay are shown. Fluorescence DiI is taken up into amphid (head) and phasmid (tail) neuron cell bodies (denoted by brackets).

Supplementary Table 1:

The list of ciliary candidate genes from *C. elegans* scRNA-seq.

Supplementary Table 2:

The human scRNA-seq list of ciliary candidate genes from Carraro, Reyfman, Habermann, Murthy, and Trachea. Indeed, ciliary genes are indicated. Carraro, Reyfman, Habermann, Murthy, and Trachea are compared and shown in the gene list. The number of shares and the names of the studies were provided.

Supplementary Table 3:

The Gold standard ciliary gene (GSCG) list was acquired from Vasquez et al (55).

Supplementary Table 4:

A negative gene list was obtained from Nevers et al (68)

Supplementary Table 5:

The list of ciliary candidate genes (only clusters 31 and 37) from comparative genomics. Known ciliary genes and ciliary candidate genes from scRNA-seq are labeled.

Supplementary Table 6:

A list of gene targets of six cilia-related transcription factors (TF), including RFX2, RFX3, MYB, GLIS3, JAZF1, and FOXJ1, is shown. The known ciliary genes are marked.

Supplementary Table 7:

Top candidate genes from the protein-protein interaction (PPI) data for IntAct, BioGRID, and HuRI (score >0.7). PPI's top candidate genes are compared to a list of negative and gold standards. All genes (11229 genes) from PPIs are provided.

Supplementary Table 8:

List of genes from text mining from Protein Atlas. The known ciliary genes are labeled yes.

Supplementary Table 9:

The top 500 genes identified by CilioGenics have been provided, and for the complete list along with scores, you can download the data from the following link: https://ciliogenics.com/

Supplementary Table 10:

The list includes the names of the genes as well as the number of articles that have identified them as ciliary candidate genes. The publication names are shown. Documentation on chosen articles is supplied, including publication year, file names, organism kinds, and paper webpage.

Supplementary Table 11:

The organism names used in comparative genomics are listed. Additionally, the protein sequences for each organism are retrieved from NCBI, and download links are provided.

Supplementary Table 12:

Sequences of primers used for genotyping and sgRNA sequence are shown.
